# Giants on Clay Feet—COVID-19, infection control and public health laboratory networks in England, the USA and (West-)Germany (1945–2020)

**DOI:** 10.1093/shm/hkac019

**Published:** 2022-06-17

**Authors:** Claas Kirchhelle

**Keywords:** Public health laboratories, Epidemic Intelligence, Preparedness, Laboratory Infrastructures

## Abstract

In early 2020, COVID-19 exposed differences in public health laboratory systems’ testing abilities. Focusing on Germany, the USA and the UK between 1900 and 2020, this article argues that studying the distinct evolution of laboratory infrastructures is critical to understanding the history of infection control and the limits of template-based reforms in global health. While each analysed laboratory infrastructure was shaped by a unique national context, neoliberal visions of lean public services and declining resources led to significant reform pressure from the 1970s. The US Center of Disease Control’s model of epidemic intelligence provided an attractive template to integrate resources and focus planning on preparedness scenarios. It also helped justify cuts to local laboratory infrastructures. Effects were uneven: in the USA and the UK, improved integration failed to compensate for local laboratory cuts and loss of autonomy. By contrast, Germany’s subsidiary principle allowed for limited federal integration while leaving local services mostly intact.

The reliable functioning of public health laboratories is a cornerstone of modern disease control. This became particularly obvious amidst the early spread of severe acute respiratory syndrome coronavirus 2 (SARS-CoV-2). Until the licensing of the first COVID-19 vaccines in late 2020, the classic public health tools of testing, contact tracing and isolation were the most effective means of containing the novel virus. On 16 March, World Health Organization (WHO) director Tedros Adhanom Ghebreyesus acknowledged this by calling on all nations to ‘test, test, test’.^1^ Reactions varied. To the surprise of many, the pandemic revealed weaknesses in the laboratory systems of USA and the UK—the two countries considered best prepared by international reviews—while lower-ranked Germany was praised for rapidly upscaling testing.^2^ In trying to explain these discrepancies between countries with similar political systems and resource levels, journalists have highlighted funding cuts for Anglo-American laboratory systems,^3^ flawed decision-making by the Johnson and Trump administrations, and errors by public health agencies.^4^ Social scientists have focused on wider structural factors such as public health responses’ lack of sensitivity towards entrenched socio-economic disparities, ineffective communication, and an over-reliance on outsourcing testing.^5^ Meanwhile, historians have described the global COVID-19 response as a sign of the fragility of biomedical promises of microbial control and the dangers of over relying on mathematical modelling and preparedness scenarios.^6^

This article complements and goes beyond these analyses by using COVID-19 as an opportunity to survey the longer-term technological trends, epidemiological ideals and political trajectories shaping public health laboratory systems in (West-)Germany, the UK and the USA. Drawing on Susan Ley Star and Karen Ruhleder’s seminal work on invisible infrastructures that become visible in times of crisis,^7^ it argues for a broader (re-)engagement with the mundane technical systems underpinning modern public health. In the case of the laboratory, historians of science and medicine have already devoted significant attention to the early history of public health microbiology and international standardisation.^8^ Meanwhile, scholars of epidemiology and public health have highlighted how rising amounts of microbial data informed new approaches to modelling disease and vaccine politics.^9^ However, few have focused on how these developments fed back into existing laboratory networks or how the later twentieth century gave rise to a new form of networked laboratory infrastructure capable of processing tens of thousands of samples and exchanging information—almost—in real time.^10^ Adaptations and gaps within these new laboratory infrastructures have similarly failed to attract sustained historical analysis. Social scientists and historians of global health have rightly highlighted complications resulting from the simplistic export of templates of laboratory surveillance, epidemic intelligence and preparedness to low-income settings.^11^ However, the fact that the very same high-income laboratory infrastructures that underpinned these developmental templates were themselves experiencing problems when it came to routine disease control has not been sufficiently reflected.^12^ As this article will show, paying close attention to the varying evolution of laboratory infrastructures is key to understanding the complex interplay between local and national disease surveillance as well as the ambivalent public health legacies of twentieth century templates of epidemic intelligence and preparedness.

After providing an overview of the unsystematic origins of public health laboratories in all three countries, the article’s first part traces the factors shaping the evolution of (West)-German, the UK, and the US laboratory networks between ca. 1930 and 1970. During this period, laboratories in all three countries became integrated into increasingly sophisticated networks of microbial processing, storage and exchange. Each network evolved to meet the needs of distinct biosecurity concerns, political environments national health systems, as well as in response to competition from commercial or hospital-based laboratory services. However, a shared underlying ideal centred on integrating the national provision of specialised reference services with routine microbial testing and public health work at the local level.

Coordinating these laboratory networks and the growing amount of microbiological data produced by them required significant fiscal investment and new forms of information processing. During the 1950s, the US Center of Disease Control (CDC) responded to the challenge of coordinating autonomous state laboratory networks by developing a new organisational template of epidemic intelligence and integrated disease reporting. Rather than producing high volumes of microbiological data, the focus was on pooling, sorting and transforming various streams of information on disease prevalence into actionable intelligence.

Part two of the article traces the domestic adoption and subsequent spread of the US epidemic intelligence template to other countries. Starting in the 1970s, authorities in the USA and the UK used the template to justify focused investment in high-tech laboratories for individual pathogens, automated sample processing, electronic reporting systems, centralised epidemiological coordination and rapid response capabilities. Reforms were, in part, driven by visions of real-time surveillance and a strategic reorientation of infection control towards hypothetical preparedness scenarios. However, in both countries, they were also a reaction to neoliberal ideals of lean public services, declining resources for routine communicable disease control, and cuts to public health laboratory services. The result was a growing imbalance of national capabilities, an outsourcing of laboratory services to the private sector and a relative loss of influence on the part of traditional public health microbiologists.

Focusing on the 1990s, part three reconstructs how their growing expertise in molecular typing, electronic reporting and epidemic intelligence allowed centres of excellence like the American CDC and the UK’s Colindale campus to serve as international models for a cost-effective, sentinel-based laboratory approach to microbial disease surveillance. It simultaneously tracks how outside of these centres of excellence, streamlined reporting capabilities not only failed to compensate for, but also helped justify further cuts to local public health infrastructures and routine diagnostic services. As described in part four, the growing structural weaknesses of Anglo-American laboratory infrastructures were not fixed by brief funding surges in response to public health emergencies. They were also not picked up by international preparedness reviews that had been shaped around ideals of pandemic preparedness and epidemic intelligence and were dominated by experts from both countries.

In all of this, (West-)Germany provides an interesting contrast to the Anglo-American history of central integration and local erosion. Following the public health establishment’s close involvement with Nazi racial hygiene, the post-war years saw competencies handed back from the federal centre to state and communal authorities. Despite Cold War competition over health indicators, public health remained a relatively neglected policy area and the sub-par performance of West Germany’s fragmented laboratory services served to highlight the advantages of more integrated systems. A major push to strengthen and integrate public health and laboratory systems occurred in the wake of reunification. Advised by the US experts, Germany began to integrate communal and state-based laboratory networks and (re-)establish the Robert Koch Institute (RKI) as a coordinating hub of epidemic intelligence. Despite this push for centralisation, the subsidiary principle (*Subsidiriatätsprinzip)* of Germany’s federal constitution, which holds that decisions should be made at the most decentralised level, meant that states continued to have considerable influence and that powers for Berlin came at the price of federal resources for local and state authorities and laboratories. This system not only helped retain local testing capabilities, but also allowed for a certain flexibility of responses should national centres of excellence be overwhelmed. The article concludes by reflecting on what insights the technical and political history of laboratory infrastructures and surveillance ideals may hold for post-COVID infection control.

## Part One: From Patchwork to Control (ca. 1930–1970)

Public health laboratory infrastructures have always been shaped by a mix of politics and science. While the roots of modern microbiology date back to the mid-nineteenth century, it was only after 1900 that bacteriological investigations began to play an increasingly prominent role in public health.^13^ The reasons for this are manifold: new technologies such as selective media, biochemical and serological typing and sero-diagnostics were enhancing bacteriologists’ ability to survey microbial environments. Collaborative investigations with epidemiologists highlighted previously unknown modes of disease transmission and dynamics as well as new environmental, animal, and human reservoirs of disease.^14^ Meanwhile, governments in North America, Europe and parts of Asia and South America were investing more resources in containing microbial threats to their civilian and military populations as well as colonial work forces.^15^

Key to the resulting ‘hunt’ for microbial reservoirs of disease was the integration of laboratory-based bacteriological with clinical and epidemiological resources.^16^ While early efforts were characterised by ad hoc alliances between different academic and medical disciplines,^17^ the First World War saw a dramatic expansion of integrated microbial monitoring. Between 1914 and 1918, all major powers deployed a mix of sanitary measures, vaccination, epidemiological surveillance, and bacteriological mass-testing to curb the spread of bacterial diseases like typhoid. Wartime bacteriologists, however, proved powerless when it came to explaining—let alone containing—the viral Spanish Flu pandemic (H1N1) or phenomena such as the variation of virulence among isolates from the same bacterial species. Meanwhile, intrusive wartime surveillance systems proved unpopular amongst civilians.^18^

Although many wartime surveillance systems were dismantled after 1918, they continued to influence the thinking of interwar planners. In all three analysed countries, the 1930s saw growing state involvement in medical and social care, rising clinical demand for testing and fears of bacteriological warfare led to a renewed emphasis on expanding and integrating public health laboratories. As will be shown, emerging laboratory networks were shaped both by unique national circumstances and the shared ideal of integrating the local provision of microbiological services with specialised national reference centres, which could identify rare pathogens, store and provide microbial reference strains, establish and enforce standardised laboratory protocols, and survey disease prevalence trends.^19^

In the UK, public health laboratories had begun to arise unsystematically at the local level since the late nineteenth century. Although calls for a cohesive national pathology service dated back to 1913, Britain’s Ministry of Health (est. 1919) and Medical Research Council (MRC) (est. 1913) only slowly began to consolidate preventive and research activities during the interwar period.^20^ A limited amount of central testing and epidemiological coordination of outbreak responses was conducted by London’s Lister Institute and a three-man Ministry of Health laboratory on Endell Street, which was ‘depressing in [its] appearance and poorly equipped’.^21^ Beyond this, most investigations continued to be carried out in a decentralised fashion by local medical officers of health (MoHs), a few dedicated communal public health laboratories, and commercial, hospital and university laboratories—who did not always report findings to the responsible MoH.^22^ Routine testing was limited, mass bacteriological sampling usually took place in response to larger outbreaks, and epidemiological analysis mostly followed 19th shoe-leather case tracking.^23^ In 1939, an incomplete survey counted at least 32 county or municipal labs across England and Wales.^24^

Biosecurity fears triggered a gradual move towards more systematised laboratory coverage. Concerns about the weaponisation of bacteriological agents had existed since the emergence of pure culture techniques around 1880. However, they were heightened during the 1920s as a result of German agents’ wartime attempts to infect Allied livestock and post-war bioterrorism allegations against Irish Republicans.^25^ Fears that Britain’s fragmented laboratory system would not be able to respond to new forms of bacteriological sabotage and warfare were voiced by the Committee of Imperial Defence in 1934 and led to the establishment of a Subcommittee on bacteriological Warfare in November 1936. In the case of war, it was decided to establish a centralised emergency laboratory network spanning urban and rural areas across England and Wales (Scotland, Northern Ireland, and London had distinct arrangements). The Emergency Public Health Laboratory Service (EPHLS) would protect troops and civilians from epidemics and bacteriological sabotage. A ‘sister’ organisation at Porton Down would develop offensive and defensive capabilities.^26^

**Fig. 1:**
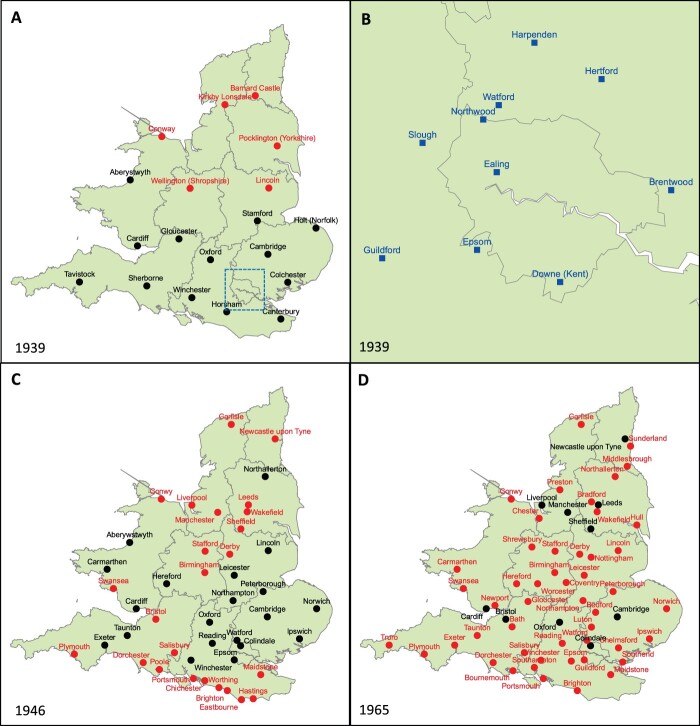
Development of (E)PHLS laboratory network 1939–65 (A) EPHLS Lab Network in 1939 (black: main laboratories; red: associated laboratories); (B) Laboratories in London sector under directorship of the Emergency Medical Service (EMS); (C) PHLS Lab network at time of PHLS foundation in 1946 (black: regional laboratories; red: area/local laboratories); (D) PHLS Lab network in 1965 (black: regional laboratories; red: area/local laboratories) Source: Williams, *Microbiology for the Public Health*; Howie, *The Public Health Laboratory Service*).

The EPHLS was mobilised as part of the newly centralised Emergency Medical Services 1 week before the declaration of war on 3 September 1939. An integrated network of 19 laboratories was set up across England and Wales with provincial laboratories reporting back to a central laboratory headquartered away from enemy aircraft in Oxford (Figure 1). Despite complaints from commercial laboratories, the ability of EPHLS to offer free epidemiological, microbiological and other laboratory services soon convinced MoHs and health professionals to collaborate with the new organisation. The collaboration held mutual benefits. At the local level, disease control was boosted by free EPHLS laboratories and experts. Standardised bacteriological data and processing (rising from 929 to 247,214 specimens per month between October 1939 and December 1941) helped EPHLS researchers survey national microbial prevalence and uncover interlinked outbreaks that would have remained invisible to local investigators.^27^

In the absence of bacteriological attacks, EPHLS research also developed new laboratory technologies: national phage-typing schemes were rolled out for typhoid, paratyphoid, *Salmonella enterica serovar* Typhimurium and *Staphylococcus aureus*; ‘Moore swabs’ were developed to isolate and trace bacteria in sewer and water systems; centralised ‘finger print registries’ for typhoid carriers were established; and microbial taxonomies were refined.^28^ Its track record secured the EPHLS a post-war future. Although Britain’s National Health Service (NHS) (est. 1948) divorced public health from medical care and maintained its own microbiology services, the renamed Public Health Laboratory Service (PHLS, est. 1946) managed to maintain a large-scale laboratory network. Many PHLS laboratories’ location in hospitals and close ties to MoHs allowed for rapid and flexible responses to local problems. Between 1947 and 1953, the PHLS expanded from 28 to 50 laboratories (Figure 1). Headquartered in Colindale, it also began to play a prestigious international role through managing international reference laboratories for *Salmonella* and *Staphylococci*.^29^ In 1960, the PHLS Act transferred responsibility for the PHLS from the MRC to the Ministry of Health—thereby establishing the PHLS as a specific body within Britain’s health protection structure.^30^

In marked contrast to parallel developments in the US, epidemiology was not a dominant discipline within the early PHLS. Despite having conducted interwar research in experimental epidemiology,^31^ the initial co-directors of the service (Graham Selby Wilson, William MacDonald Scott, and William Whiteman Carlton Topley) had focused the service on providing basic and specialist laboratory services to MoHs. Resulting epidemiological outbreak investigations were usually headed by MoHs with PHLS specialists advising. The focus on microbiological service provision was mirrored in the wider organisational structure of the PHLS, which centred on relatively autonomous local laboratories and a select number of powerful reference laboratories in London. The service’s main monthly bulletin remained dominated by articles on laboratory techniques and protocols, the identification of new bacterial types, and occasional model investigations of individual outbreaks.

A dedicated PHLS Epidemiological Research Laboratory was eventually established under the directorship of W. Charles Cockburn in 1946. Similar to the American CDC, Cockburn’s office began to gather national disease data—first on salmonellosis and then on other diseases—and provided expertise on the efficacy of pertussis vaccine roll-outs and clinical trials involving experimental polio and rubella treatments and prevention. A weekly joint publication of microbiological and epidemiological information began to be published from the late 1950s onwards.^32^ However, overall, epidemiology continued to play a secondary role to public health microbiology.

Parallel developments in the USA would transform this relatively passive mode of national epidemiological analysis into a new template of active epidemic intelligence centring on integrated data gathering, centralised analysis, and rapid outbreak response capabilities. The new template would not only shift the relative influence of public health microbiologists and epidemiologists in favour of the latter group, but also prove a successful international export.

Similar to the UK, the US public health laboratories had emerged in an unsystematic fashion from the late nineteenth century onwards. In 1887, a National Hygienic Laboratory (predecessor of the National Institutes of Health) was established at the Marine Hospital in Staten Island. By 1907, all the US states had boards of health, which were supplemented by local and county health departments.^33^ In 1914, 47 of 48 states reported on the activities of their dedicated public health laboratories. National organisations like the State Laboratory Directors Conference (est. 1927, now Association of Public Health Laboratories (APHL)) and Association of State and Territorial Health Officials (est. 1942) were established to coordinate activities and large-scale Rockefeller Foundation funding for disease control further boosted laboratory capacity.^34^

However, in a noted difference to England and Wales, responsibility for infection control was not transferred to the federal level but continued to rest with individual states. This meant that federal officials focused efforts on strengthening inter-state surveillance and assisting state-level programmes with earmarked funds and expert services.^35^ During the interwar period, the Public Health Service (PHS) and other federal authorities stepped in to stop human, animal and plant diseases from crossing state borders. The PHS also supported states with the control of venereal disease and malaria and collected data on 29 notifiable diseases.^36^ Legislative reforms increased ties between federal and state authorities. The 1935 Social Security Act provided a permanent machinery for the distribution of federal funds via grant-in-aid to state and larger local health departments and the 1938 National Venereal Disease Control Act released significant funds for state-based interventions.^37^ However, ultimate authority over laboratories and funding continued to rest with individual states.

Despite improved interwar coordination, state-based responsibility meant that there remained considerable variation in the quality, number and speed of laboratory services. Free official public health laboratory services were unevenly distributed with poorer and rural areas enjoying less coverage than wealthier and metropolitan areas. Responding to these gaps, a growing number of commercial mail order and hospital-based pathology laboratories offered for-profit tests for various diseases. Quality concerns led to attempts by professional societies to regulate commercial services via approved laboratory lists and training curricula.^38^ Quality assurance problems were not limited to the private sector. In so-called centralised states, state health authorities operated local health departments as branches, which made laboratory quality control and data collection easier. However, in decentralised states, local health departments were financed by local governments and retained more authority and responsibility for the delivery of public health services, which complicated oversight.^39^ Resulting variations in data provision and quality made it hard to make robust claims about national or state-level disease incidence.

Problems were compounded by blurred state-level responsibilities. In 1940, a PHS survey came to the ‘somewhat startling’ realisation that ‘within a single State as many as 18 separate agencies contribute something to the health activities’ and that ‘no jurisdiction’ had less than ‘6 agencies involved’.^40^ Communicable disease responsibility was mostly located within state health departments, but additional responsibilities could be located in as many as eight other agencies. Blurred responsibilities negatively impacted notification practices and laboratory quality control.^41^ Laboratory work was also heavily skewed towards a limited number of prominent pathogens. As a result of the 1938 Venereal Disease Law and marital examination laws, syphilis testing occupied two-thirds of US states’ entire diagnostic laboratory work. However, only 16 states regularly assessed commercial Wassermann tests and only nine controlled diagnostics for other diseases.^42^ Funding differences exacerbated performance variations. In 1940, US states spent ca. $285 million on public health (ca. $1.90 per capita). However, Tennessee only spent $0.76 per inhabitant whereas New York spent $3.27 and Hawaii $5.03.^43^

For the US infectious disease control to improve, there was a need to boost coordination between federal and state authorities, expand laboratory services and standardise laboratory testing and reporting protocols. Similar to the UK, the Second World War provided an important stimulus. Domestically, disease control was an important part of wartime efficiency drives and justified investment in public health and laboratories systems. Externally, the global nature of the war and concerns about Japanese biowarfare justified creating centralised epidemiological coordination and medical intelligence capabilities.^44^ Both developments carried over into the post-war period. With spending increasing at the local, state and federal levels, 86 per cent the US citizens were served by a local health department in 1950 and over 34,000 persons were employed full-time in various public health agencies.^45^ The 1944 Public Health Service Act also strengthened central oversight by specifying a federal coordinating role over US public health.^46^ From 1951, PHS officials and the Council of State and Territorial Epidemiologists began to meet annually to decide on a list of diseases to be reported by states. Notified data was soon compiled and published on a weekly basis in what would become the *Morbidity and Mortality Weekly Report.*^47^

The biggest innovation to come out of the 1940s reordering of the US public health was a national centre of epidemiological and bacteriological expertise. Originating in wartime malaria and typhus control programmes, the CDC was established as the Communicable Disease Centre in 1946. As an initially obscure federal department outside Washington DC, the young agency had to convince Congress, the PHS, and individual states to finance its activities and transfer powers.^48^ Another challenge lay in expanding its remit from vector-borne to other diseases. Boosted by biological warfare concerns and expanding vaccine schedules,^49^ the CDC solved both challenges by providing epidemiological advice, bacteriological reference services and rapid local assistance during outbreaks. Between 1950 and 1960, the young agency acquired responsibility for federal disease responses (1950), established a national network of assigned typing laboratories (1951), acquired responsibility for venereal disease (1957) and tuberculosis control (1960), and oversaw programmes against polio (1955), influenza (1957) and nosocomial infections (1957).^50^ The 1969 Clinical Laboratories Improvement Act further expanded CDC power over domestic microbiology by giving it the authority to license clinical laboratories soliciting or accepting specimens in interstate commerce. Internationally, the CDC turned into an increasingly prominent arm of the US soft power diplomacy and was designated International Influenza Center for the Americas, International Shigella Centre, and WHO serological reference centre for syphilis.^51^

Although some have described it as a ‘dumping ground’^52^ for declining public health programmes, the rapid expansion of the CDC was underpinned both by bureaucratic opportunism and an increasingly concrete ideal of centralised epidemic intelligence. Because it never acquired control over state or local laboratory networks, the CDC focused on supporting states and gathering and analysing resulting disease data. This focus on supporting rather than controlling baseline public health services was reflected in its motto: ‘We exist to serve the states.’^53^ It also led to a distinct distribution of power between the agency’s four branches (epidemiology, laboratory, training and technology). In Savannah, the CDC’s technology branch acquired significant resources through its involvement with the US malaria eradication programmes and by developing equipment for mass vaccination campaigns. In Atlanta, the training branch provided courses and educational material while the bacteriology branch gained international renown through the work of microbiologists like Philip R. Edwards. However, it was in the epidemiology branch that the biggest influence and power within the CDC rested.^54^

Headed by famed epidemiologist Alexander Langmuir, the CDC epidemiology branch built on wartime models of ‘medical intelligence’ by conceiving of infectious disease surveillance as a form of systematised intelligence gathering and analysis. In contrast to the traditional focus of public health surveillance on tracing the transmission of pathogens between individuals, Langmuir focused on surveying larger (human and microbial) populations.^55^ According to Langmuir, surveillance was:

… the continued watchfulness over the distribution and trends of incidence through the systematic collection, consolidation and evaluation of morbidity and mortality reports and other relevant data. Intrinsic in the concept is the regular dissemination of the basic data and interpretations to all who have contributed and to all others who need to know. The concept, however, does not encompass direct responsibility for control activities. These traditionally have been and still remain with the state and local health authorities.^56^

Although Langmuir was careful not to infringe on state competencies, this did not mean that he thought of surveillance as a passive activity. At his behest, a dedicated CDC Epidemic Intelligence Service (EIS) was formed in 1951. With deliberate overtones of military intelligence activities, the EIS was tasked with coordinating responses to disease outbreaks—and biological attack—that exceeded local and state capabilities. A cadre of EIS officers could rapidly deploy while Atlanta provided centralised coordination. The EIS’ logo summed up this combination of ‘boots on the ground’ field epidemiology, centralised analysis and increasingly international ambition by depicting a globe straddled by a sole.^57^ Despite fierce rivalries with other CDC ‘fiefdoms’^58^—in particular the laboratory branch—Langmuir’s vision of integrated surveillance and epidemic intelligence would play a profound role in shaping the organisational evolution of the CDC and wider international thinking about infection control.^59^ Whether the existing organisation and scale of post-war public health laboratory infrastructures was the most cost-effective way of achieving this control became debatable.

In contrast to Britain’s centralised laboratory infrastructure and the US model of state-based systems and service-like federal coordination, West Germany’s public health laboratory infrastructure was deliberately decentralised. During the *Kaiserreich*, German bacteriology had achieved international renown but remained decentralised outside of Prussia and the military. By 1911, there were over 50 official bacteriological institutes across the German states.^60^ Centralisation efforts occurred during the First World War and the interwar era. However, it was the fusion of public health and racial hygiene during the Nazi era that triggered the most dramatic expansion of state investment in and centralisation of public health activities. Within a year of the 1934 law for the unification of the health system (*Gesetz zur Vereinheitlichung des Gesundheitswesens*), there were 740 official public health departments (*Gesundheitsämter*) across Germany. Local and state-level departments and hygiene institutes frequently employed bacteriologists to screen populations for venereal disease, tuberculosis, and other perceived threats to public and racial health*.* There were also close connections between leading bacteriologists at the *Reichsgesundheitsamt*, the military and the *RKI* at the national level.^61^ A further intensification of laboratory and clinical screening occurred following the passage of the regulation to fight transmissible diseases on 1 December 1938—although wartime pressures soon constrained available resources.^62^

Their enthusiastic support of Nazi racial hygiene meant that large parts of the German public health and bacteriological establishment fell into disrepute after 1945. In West Germany, prime responsibility for public health laboratories and infection control was decentralised and firmly re-embedded in the Federal Republic’s 10 states (*Bundesländer*) and powerful municipalities (*Kommunen*).^63^ In contrast to communist East Germany’s centralised health and public health systems, West Germany’s federal government limited itself to acting as a political ‘manager’ enacting compromises between different parts of a complex health system but developed little epidemiological or bacteriological expertise of its own. The wider West-German health system consisted of ca. 500 local and communal health offices (*Gesundheitsämter*)—often with their own public health laboratories—10 state health departments (*Landesgesundheitsämter*), over 1,300 statutory health insurers covering about 90 per cent of the population, and powerful practitioner and hospital associations.^64^ Federal authority for public health rested with the Federal Office of Health (*Bundesgesundheitsamt*),^65^ the Federal Ministry of Health (*Bundesgesundheitsministerium*, est. 1961), and the Conference of Health Ministers of the Federal States (*Gesundheitsministerkonferenz*). Below the federal level, some states like Bavaria had integrated public health laboratory services while others relied on communal arrangements for laboratory provision with loose coordination via state health departments.^66^

Fragmented responsibilities and the divorce of state-funded public health from insurance-funded curative medicine led to a long-term stagnation of the former.^67^ By the 1970s, relative oversupplies of hospital beds and doctors contrasted with withering investment in West German public health and laboratory capacity.^68^ Despite strong inter-German rivalry over health indicators and expanding vaccination schedules, West Germany did not systematically boost or integrate laboratory networks and epidemiological monitoring.^69^ According to a 1985 WHO-commissioned review, there was ‘virtually no planning explicitly directed towards improving the health of the population, improving the effectiveness and quality of health care, or intensifying protection against illness’.^70^ Although the number of public health positions per 1,000 inhabitants remained stable on paper, low salaries and prestige led to recruitment problems and a significant number of vacancies.^71^

While some reviewers noted that West German decentralisation created a certain ‘flexibility’,^72^ lack of integration and the relative insularity of post-war West German epidemiology and bacteriology negatively impacted disease control.^73^ There was limited coordination at the federal and state level via stakeholder conferences and circulars.^74^ Local public health laboratories could request specialist assistance from universities, the RKI (part of the Federal Health Office from 1952 to 1994), or Hamburg’s Bernhard Nocht Institute for Tropical Medicine. However, health sector rivalries and lacking federal interest meant that infectious disease control as an integrated national endeavour remained neglected. The Federal Health Ministry only began publishing internationally comparable disease statistics in 1963, there was no regular and comprehensive assessment of health statistics, and no West German institution could rival the PHLS or CDC as an international authority for laboratory standards, public health training, research and epidemic intelligence.^75^

## Part Two: Neglect (1970–1990)

The ongoing post-war decline of infectious disease led to a relative decline of attention for traditional pathogen control and surveillance in all three analysed countries. Although Cold War competition and international health programmes strengthened the influence of the PHLS and CDC over laboratory systems in other parts of the world,^76^ public health managers had to contend with a shift of resources away from laboratory-based pathogen surveillance towards chronic and non-communicable diseases.^77^ The described shift of attention towards non-communicable diseases coincided with a wider economic reordering of public health. In line with contemporary governments’ attempts to control rising expenditure, managerial cost–benefit thinking and neoliberal ideals of ‘lean’ government led to a restructuring of public health laboratory infrastructures. New technologies like automated sample processing promised to make laboratory systems more efficient whilst simultaneously allowing for personnel cuts. Meanwhile, the CDC’s model of epidemic intelligence provided an attractive template to justify the pruning of existing laboratory services. In contrast to 1930s visions of public health laboratory infrastructures with a strong local presence and relative autonomy regarding sampling remits, planners increasingly focused investment on epidemiological hubs and integrated specialist laboratory networks for the surveillance of a select number of important pathogens. The goal was to transform public health surveillance from a mostly ‘archival function’ to a streamlined system ‘in which there is timely analysis of the data with an appropriate response’.^78^

In the UK, the 1974 NHS reforms transformed public health. Since 1939, close local collaborations between relatively autonomous PHLS labs, MoHs and clinicians had been a hallmark of British public health.^79^ However, in 1974, the once powerful office of the MoH was disbanded. With Britain’s welfare state and costs expanding, the 1974 reforms aimed to improve the management, quality, and integration of social and health services by transferring the responsibilities of local authorities for personal health services outside hospitals to new NHS regional and area health authorities.^80^ MoHs’ importance had already declined as a result of GPs’ expanding role in maternal and child health and the removal of social care from their remit. Their remaining duties were now distributed among other local authority and NHS services.^81^ The move fragmented and undermined community-level public health.^82^ Local authorities retained environmental and some public health services, GPs remained responsible for maternal and child health, and community physicians with ill-defined preventive health duties were appointed to NHS regional, district, and area authorities.^83^ According to later estimates, the 1974 reorganisation also led to a significant loss of expertise by prompting the early retirement or dispersal of ca. 400 highly skilled public health doctors.^84^

The severance of traditionally close ties between local public health laboratories and MoHs and growing tensions between public health and clinical microbiologists also impacted the PHLS. Since 1946, the PHLS laboratory network had continued to expand before reaching its maximum extent in 1969. A large part of this growth was achieved by establishing dual purpose laboratories that were embedded in NHS hospitals and provided both public health and clinical microbiology services. By 1969, 42 of 63 PHLS constituent laboratories were providing general bacteriological services to the hospitals they were based in, 13 were supplying some kind of microbiological service to a hospital, and only 8 were solely conducting public health research.^85^ The advantages to the PHLS were obvious: clinical microbiology generated revenue and data on infection levels, enabled close working relations with local clinicians, and facilitated outbreak responses. However, existing arrangements also made NHS clinical pathologists accuse the PHLS of ‘poaching’ their work.^86^ Around 1966, it was agreed that the PHLS should only provide services to local hospitals if invited to do so.^87^ Three years later, a review of PHLS activity recommended against further merging clinical and public health microbiology. Public Health Laboratory Service core tasks were now defined as ‘to make a continuous study of how microbial diseases are spread, and what advice should be given for their control’.^88^ The PHLS would remain present in British hospitals. However, it would have to close 13 of its 63 laboratories over the next 10 years (see Figure 1). Coupled with the 1974 loss of access to MoHs, this was a first blow to the 1939 vision of public health laboratory services that were closely integrated with local epidemiological and clinical expertise.

Although the PHLS secured the reassignment of most its laboratory staff, the 1970s fiscal crisis and growing government emphasis on non-communicable diseases created competition for resources and pressure for cost-effective microbiology services.^89^ In response, the PHLS invested in the computerisation and automation of routine microbiological testing.^90^ PHLS authorities also tried to find ways of compensating for the loss of MoHs by developing new ways of integrating microbiological and epidemiological services. The PHLS’ fragmented response to the 1973 smallpox release at the London School of Hygiene and Tropical Medicine and concerns about imports of viral haemorrhagic fevers further highlighted the need to strengthen in-house epidemiological expertise and improve outbreak responses.^91^ A resulting review called for a national centre of epidemiological expertise.^92^

The obvious template for reform seemed to be the CDC’s model of epidemic intelligence. In 1977, Britain emulated the US agency by founding the PHLS Communicable Disease Surveillance Centre (CDSC) under the leadership of Nicol Spence Galbraith. Recruited in 1976, Galbraith had previously worked as an MoH and area medical officer in the London area and had long argued for a centrally financed and coordinated national epidemiological service along the lines of the CDC.^93^ Initially, it seemed that Galbraith’s CDSC would have a similar status to the PHLS Epidemiological Research Department with only one additional staff member and a seconded senior medical officer working in a repurposed portakabin. However, following a 1977 visit to the CDC in Atlanta, Galbraith embarked on a programme of coordinated expansion. In 1978, the CDSC achieved national prominence by quickly reacting to the 1978 Birmingham smallpox outbreak.^94^ The episode allowed the CDSC to prove its value as a ‘highly active information and co-ordinating centre’.^95^ In addition to receiving its own building (1980), it began publishing a CDC-style weekly *Communicable Disease Report* (CDR) on national prevalence levels. It also acquired competencies for the national surveillance of immunisation programmes (1985), began offering teaching and training in communicable disease epidemiology (1988), and used the resulting ‘cadre’^96^ of officers to conduct EIS-style field investigations at the behest of local authorities. However, an expansion into the area of environmental health and non-communicable diseases beyond the traditional microbiological focus of the PHLS was resisted.^97^

The rise of the CDSC as the PHLS’ epidemiological ‘nerve centre’^98^ and growing focus on the US-style epidemic intelligence coincided with a relative decline in the perceived importance of the local public health laboratories that had previously formed the backbone of the PHLS. Despite increases in the volume and integration of microbiological sampling, a growing number of voices began to question the cost effectiveness of existing public health laboratory services: the *ad hoc* nature of 1970s PHLS lab closures had increased discrepancies between a better served south and a more poorly served north; fiscal constraints were forcing the PHLS to press NHS authorities for more compensation of microbiological services; and the new Thatcher administration included the PHLS in its programme of cost cutting reviews.^99^

**Figure. 2:**
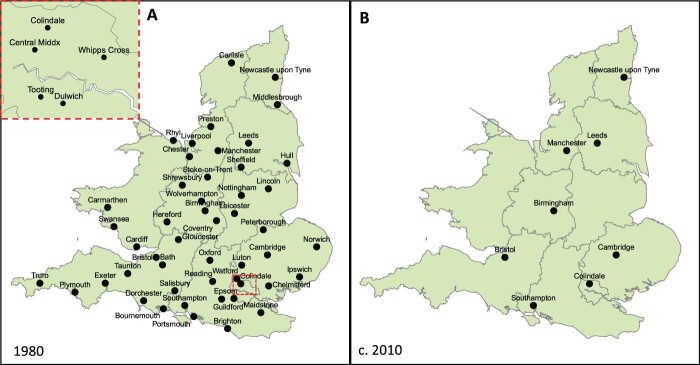
Extent of laboratory network under PHLS (1980) and HPA (c. 2010) (A) Extent of PHLS network following 1970s laboratory closures in 1980, London laboratories highlighted in box; (B) Location of HPA regional laboratories following NHS takeover of PHLS area laboratories in c. 2010. Source: Williams, *Microbiology for the Public Health*; private communication.

In 1985, a Department of Health and Social Services (DHSS) review recommended a radical transfer of the PHLS’ 52 area and regional public health laboratories (Figure 2) to NHS health authorities—a move that would have turned Colindale into a CDC-like centre of epidemic intelligence rather than a national provider of public health microbiology.^100^ The proposal prompted fierce criticism from British health professionals. Writing in the *British Medical Journal* in 1985, former PHLS director James Howie warned against demoting the PHLS to a consulting epidemiological service and breaking up a laboratory network that connected London to the rest of the country. Contrasting Britain and the USA, Howie was adamant that the ‘peripheral’ contacts provided by its extensive laboratory infrastructure made the PHLS more resilient and attuned to local problems than other systems. Planners should strengthen both epidemiological and microbiological services rather than demoting one in favour of the other:

So the PHLS is at a crossroads. Starved of funds, it can maintain its national laboratory service only at the expense of curtailing its epidemiological activities – or conversely maintain or increase its epidemiological activities only at the expense of the laboratory service. (…). What seems plain is that [NHS] district health authorities have neither the incentive nor the powers to get going measures of action and investigation covering regions wider than their own. (…). [The PHLS] is admired and envied for its effective peripheral contacts by its counterpart in the United States (…). Those who would break the essential PHLS links between centre and periphery – who are they? Do they really know what they are doing?^101^

The DHSS review’s call for strengthened central epidemic intelligence prompted a merger of the PHLS Epidemiological Research Service and CDSC (1985). However, medical criticism, rising numbers of disease outbreaks, and the contemporary HIV/AIDS crisis made the Thatcher government decide to maintain the wider PHLS laboratory network.^102^ Howie’s warnings nonetheless proved prescient: by the 1990s, budget pressures, politicised responses to major health crises and hopes that electronic integration and epidemic intelligence would be able to compensate for slimmed down local microbiology services began to pose a serious threat to Britain’s public health laboratory infrastructure.^103^

Developments in the USA highlighted the dangers of neglecting investments in routine local public health laboratory services in favour of a limited number of national and regional hubs of excellence. Internationally, the CDC remained a poster child of microbiological and epidemiological expertise due to its rapid outbreak responses, research on classic and new pathogens (e.g. cholera and haemorrhagic fevers like Lassa, Marburg and Ebola), and prominent role in the wider global smallpox eradication effort.^104^ As described by Etienne Gosselin, its attractive mix of providing assistance to states (both domestically and abroad) via technology, epidemic intelligence and field epidemiology served as an ‘interface through which the CDC penetrated international health programs and institutions’.^105^

However, outside of Atlanta, US politicians’ commitment to routine infectious disease control was waning. In the fiscal year 1973/1974, there were 12,295 clinical and public health laboratories in the USA. Around 3 per cent (392) of these were official state and local public health laboratories, which performed ca. 70 million laboratory tests per year (2 per cent of total laboratory examinations).^106^ Per capita expenditure and the number of analysed public health specimens increased throughout the 1960s and 1970s.^107^ This increase was driven less by classic work on communicable disease^108^ than by toxicological, microbiological and chemical testing related to growing concerns about chronic disease and environmental health.^109^ Rising per capita expenditure also belied increasing understaffing in public health laboratories. After peaking at 6,598 in 1975, the number of budgeted positions in territorial public health laboratories declined to 5,603 (−15 per cent) in 1982.^110^ Recruitment problems meant that many budgeted positions remained unfilled with a vacancy rate of 11 per cent reported in 1979.^111^ Whereas free public health testing had traditionally been viewed as an essential service, fiscal pressure, competition from commercial laboratories and the retreat of universities from unlucrative public health work made an increasing number of public health laboratories charge fees. Additional problems resulting from lack of coordination, underinvestment in laboratory improvement and ‘red tape’ prompted some commentators to call for an abandonment of all free routine testing and a concentration of public resources on specialist laboratories and microbial reference services.^112^ For many decision-makers, the broad local and state public health laboratory services that had helped lay the foundations for the rise of US epidemic intelligence were no longer essential.

Initially, it seemed as though the CDC in Atlanta might escape some of these domestic pressures. Promoted from the rank of PHS Division to Bureau in 1968, the agency profited from rising health spending by the Johnson and Nixon administrations and acquired new competencies in areas unrelated to communicable disease including nutrition, occupational safety, smoking and environmental health.^113^ The new areas of involvement were a reaction to the contemporary epidemiological transition and declining political attention for infectious diseases. They also diverted agency resources away from traditional public health microbiology towards the epidemiological assessment of risk factors associated with lifestyles, diets and environmental factors. To reflect its shift away from communicable disease, the agency was renamed Center for Disease Control in 1970.^114^

The CDC’s expansion into new areas was not without challenges. After moving from success to success during the first decades of its existence, the 1970s saw the CDC experience a series of setbacks and financial pressures. Starting in 1974, successive US administrations responded to surging inflation and economic stagnation (stagflation) by imposing limits and cuts on federal expenditure. In 1976, the CDC suffered additional damage due to its advocacy of mass vaccination against swine flu (H1N1), which resulted in the sacking of its long-standing director David J. Sencer and major organisational reorganisation. In line with the Surgeon General’s 1979 call for a second public health revolution centring on preventive health, the CDC was renamed into Centers for Disease Control and Prevention and focused more of its increasingly constrained resources on lifestyle and environmental issues.^115^

Following Ronald Reagan’s election, Sencer’s successor William Foege was forced to administer severe cuts of federal public health funding.^116^ Once again, microbiological expertise did not fare well. Despite the parallel emergence of HIV/AIDS in the USA, the CDC announced it was considering laying off between 350 and 780 of its 3,700 employees in 1982 with about half of lay-offs planned in the area of infectious disease.^117^ Between 1981 and 1983, the CDC’s budgeted staff-year—or full-time equivalent workload—ceiling sunk from 4,249 to 4,045 years (−4.8 per cent). Further cuts between 1985 and 1988 reduced the ceiling for original departments to 3,904 staff years (−8.12 per cent).^118^ Cuts further concentrated agency resources towards cost-effective epidemic intelligence and strategic response capabilities for high-priority risks.

While the USA and UK thus saw fiscal pressures, declining interest in infectious disease, and an increasing focus on centralised epidemic intelligence erode resources for local and national public health laboratory services, West German arrangements highlighted the dangers of lacking epidemiological integration and local fragmentation. Throughout the 1960s and 1970s, post-war arrangements had remained relatively static: local *Gesundheitsämter* were responsible for most laboratory testing and public health enforcement; state offices compiled data, aided outbreak responses and administered health programmes; federal activity mostly focused on the political coordination of state programmes. Epidemiological oversight was limited. According to a 1976 survey of venereal disease control, public health workers lacked power in relation to insurance-financed clinicians. Many clinicians did not adhere to updated 1970 disease reporting requirements and had monopolised lucrative treatments. The small number of local *Gesundheitsämter* offering consultative and laboratory services referred patients to local practitioners. Systematised laboratory testing was limited, disease incidence was under-reported, and data was insufficient to allow for effective contact tracing.^119^ Public health’s low status and the absence of comprehensive clinical and microbiological data made it difficult to apply new concepts of epidemic intelligence, recruit talented workers or convince politicians of the value of public health investments.^120^

Post-war resistance of the West German states to federal centralisation gradually diminished from the 1970s onwards. However, attempts to update disease reporting, improve integration and expand coverage with 1972 ‘guidelines for federal state law on public health’ and 1977 ‘Concerted action in the public health sector’ failed to resolve problems.^121^ There were limited updates of state health service provisions and dental and preventive services were rolled out to wider population segments.^122^ However, key responsibilities such as disease screening were assigned not to the *Gesundheitsämter* but to ambulatory care physicians, who were reimbursed by sickness funds. This further diluted officials’ capacity to expand, collate and act on laboratory testing data. It also eroded the wider public health workforce. Between 1970 and 1996, the number of physicians working in West German public health services decreased from 4,900 to 3,300, the number of dentists from 2500 to 800, and the number of social workers from 4,000 to 2,500.^123^ Without epidemiological coordination and the integration of state and communal networks, West German public health laboratory infrastructures remained characterised by fragmentation, localism and poor morale.

Lack of political interest and planning towards ‘intensifying protection against illness’^124^ was also reflected in poor scientific guidance coming from the federal centre. In 1975, the *Bundesgesundheitsamt* recruited 50 experts to develop recommendations on nosocomial infections. However, in contrast to contemporary Anglo-American programmes, resulting guidelines on architecture and technical procedures did not cite systematic reviews or clinical studies.^125^ External reviews of West Germany’s public health system were harsh. A 1991 assessment found services to be both ‘highly decentralised and highly formalised’—public health services were ‘weak’ and ‘something of a poor relation’.^126^ Immunisation rates were comparatively low and—despite the significant gaps characterising disease and laboratory reporting—infectious disease mortality (excluding HIV/AIDS) was over 40 per cent in excess of socialist East Germany.^127^

By the end of the 1980s, post-war laboratory infrastructures had thus evolved in very different directions. In both the USA and the UK, budgetary pressures, a redirection of attention away from infectious disease, and visions of integrated epidemic intelligence had strengthened central epidemiological oversight but weakened local public health laboratory services. By contrast, lack of epidemiological oversight and integration of local and state laboratory infrastructures was hampering infection control in West Germany.

## Part Three: Clay Feet (ca. 1990–2000)

Budgetary and political pressure on post-war laboratory infrastructures accelerated during the 1990s. Pressures occurred amidst growing scientific warnings about public health systems’ ability to deal with surging rates of antimicrobial resistance (AMR), nosocomial and food-borne infections, and the accelerating HIV/AIDs pandemic. In the USA and the UK, health officials and experts tried to mobilise resources by framing infectious disease hazards as national biosecurity threats. Building on the now firmly established international template of epidemic intelligence, advocates claimed that identified threats could be tackled with improved mathematical forecasting, networked sentinel laboratories and molecular ‘real-time’ surveillance, and centralised intelligence and response capabilities. Resulting preparedness plans did not sustainably strengthen either domestic laboratory infrastructures or microbiological capacity in the foreign locations they were also targeting. Instead, they concentrated resources on high priority pathogens and individual hubs of excellence. Focusing on microbiological surveillance in Africa and South Asia, anthropologists, microbiologists, and historians have highlighted how ring-fenced, time-limited, and targeted surveillance programmes hollowed out laboratory capacity for other pathogens.^128^ The same was true in the countries pushing this agenda abroad. In the UK and the USA, investment in preparedness capabilities coincided with accelerating cuts to and the privatisation of local public health laboratory services. Germany proved an exception. Following a major scandal, the US templates of epidemic intelligence and preparedness were imported but adapted to the political realities of German federalism whereby strengthening federal powers came at the price of investment in state and communal services.

For the UK’s PHLS, the period around 1990 proved particularly challenging. During the 1980s, HIV/AIDS and fatal outbreaks of salmonellosis at a mental health unit at Wakefield’s Stanley Royd Hospital (1984) and of legionella at Stafford General Hospital (1985) had triggered a wider review of public health services. Headed by Chief Medical Officer (CMO) Donald Acheson, the review’s influential 1988 report stressed the value of epidemiological oversight and stated that the 1974 NHS reorganisation had fragmented public health at the community level. MoHs had once provided a public health anchor point for local authorities, general practitioners, PHLS laboratories and hospitals. However, after this office was disbanded in 1974, ill-defined responsibilities had led to a neglect of local preventive health and communicable disease control.^129^ This neglect had been exacerbated by 1982/1983 managerial reforms, which abolished NHS area authorities and resulted in the retirement of 20 per cent of community physicians—many of them former MoHs—recruitment problems, and low morale.^130^

In the case of infectious disease control, coordination between local PHLS laboratories, NHS services and local authorities was compromised by the absence of overarching epidemiological oversight. It was ‘extremely important (…) that someone within the health authority is responsible for linking the vital work undertaken by microbiologists and control of infection teams within hospitals with cases of infection occurring outside’.^131^ Effective infection control at the local level was also hampered by national funding and data integration problems within the PHLS:

Evidence submitted to us demonstrates almost universal support for the PHLS and its epidemiological ‘nerve centre’ the CDSC. (…). We are concerned to learn that if there were a recurrence of serious outbreaks (…) in more than one part of the country at the same time, or if a single outbreak spread to more than one major centre of population, the current system would be unable to cope.^132^

To strengthen communicable disease control, the 1988 Acheson report recommended re-embedding epidemiological expertise at the local level by creating the post of a medically qualified District Control of Infection Officer (eventually called Consultant for Communicable Disease Control (CCDC)), who would provide advice for local public health and clinical services. A second new post, the Director of Public Health (DPH), would work with the CCDC, coordinate preventive health at the district level and submit public health annual reports.^133^ CCDCs would also be part of an advisory District Control of Infection Committee and would receive district and regional NHS support for contact tracing and public health education.^134^ At the national level, the PHLS should be strengthened by making it mandatory to report outbreaks to its epidemiological service, the CDSC. In line with its focus on improving data integration, the report also called for a new national electronic disease notification system connecting local laboratories and clinics with the CDSC in Colindale.^135^

While the Acheson report revitalised community public health,^136^ government promises to create full NHS consultant posts for infection control trainees fell through and calls for a sustained strengthening of wider PHLS services went unheard.^137^ Instead of funding both the ‘nerve centre’ and its microbiological sensors, the 1990s saw the PHLS forced to absorb budget cuts to its laboratory services and prove its cost-effectiveness with value for money reviews and new NHS customer arrangements.^138^ PHLS workers also faced the challenge of updating phenotypic surveillance methods with new genotypic molecular technologies like pulsed-field gel electrophoresis (PFGE) and of replacing paper-based with electronic reporting systems without compromising patient confidentiality.^139^ In 1994, the PHLS launched a concerted effort to facilitate real-time surveillance by establishing a National Surveillance Group. The group was commissioned to review protocols for the surveillance of specific infections and launched an Epidemiology Information Technology Strategy to upgrade IT systems.^140^ New EU initiatives on transnational AMR monitoring and prospects of rapid near patient testing triggered the creation of additional surveillance systems to integrate laboratory and clinical information on infectious disease prevalence and microbial characteristics.^141^

On the occasion of its 50-year jubilee in 1996, the PHLS thus remained in direct command over a network of 49 microbiology laboratories organised into nine regional groups with devolved budgets and could also draw on information supplied by NHS laboratories.^142^ However, ongoing calls for streamlined and—more importantly—cost-effective microbiology services made the future of this formidable infrastructure seem increasingly uncertain. Critical commentators warned that no amount of centralised epidemiological surveillance and electronic integration would be able to fully substitute effective local laboratory services. Reflecting on the PHLS’ contributions, the *Lancet* warned: ‘At a time when the British government seems keen to be penny wise and pound foolish, the message (…) to those who would further pare down an already rationalised PHLS is leave well alone.’^143^

Existing public health laboratory infrastructures faced even graver pressures in the USA. The cuts in 1980s to federal and state public health budgets and attempts to commercialise microbiology services had resulted in a marked decline of public health laboratory capacity.^144^ Between 1977 and 1982, dedicated staffing in state laboratories declined by 6.9 per cent.^145^ From 1981 onwards, Reaganite cuts to new federal block grants triggered proportional cuts of state expenditure on public health administration, environmental health and laboratory provision.^146^ Public health spending also decreased at the local level.^147^ Reduced federal, state and local spending was accompanied by an increase of infectious disease incidence. Between 1980 and 1992, the US death rates from infectious diseases rose by over 50 per cent and once again became the third most common cause of death.^148^

Alarmed, the Institute of Medicine (IoM) published a damning analysis in 1988: ‘disarray of public health’^149^ had resulted from underfunding, reactive policymaking, varying state capabilities and fragmented services. All but one state conducted laboratory analyses for communicable diseases, and all screened their population for over 30 health problems.^150^ However, the diseases being surveyed and the reporting methods varied.^151^ To improve public health, the IoM recommended creating regularly reviewed state public health strategies. The US states were ‘the central force in public health’^152^ but federal and state authorities had to do more to guarantee essential services like laboratory access to citizens, expand and integrate data collection, and coordinate existing assets. Congress and the CDC should use federal funds to balance fiscal disparities between states.^153^

Co-chaired by Nobel Laureate Joshua Lederberg, a second IoM review issued an even sterner call for action in 1992. Titled *Emerging Infections: Microbial Threats to Health in the United States*, the review warned that the USA had no ‘comprehensive national system for detecting outbreaks of infectious disease (except for food- and waterborne diseases)’ and that there was a ‘lack of coordination among the various US government agencies’ and ‘between government agencies and private organisations’.^154^ To prepare for future ‘infectious disease emergencies’,^155^ federal authorities should create national stockpiles and push for a ‘comprehensive global infectious disease surveillance system’.^156^ To compensate for inconsistencies and growing surveillance gaps at the local and state level, reviewers called on federal authorities to boost epidemic intelligence and response capabilities via improved epidemiological training at state-level and integrated electronic data systems. It also recommended that the PHS establish a ‘comprehensive computerised infectious disease database’^157^ to consolidate information from domestic and international programmes.

Originating in a 1989 conference on *Emerging Viruses*, the 1992 IoM report is now seen as a landmark publication of a new age of biosecurity politics, preparedness scenarios and hopes for ‘real-time’ surveillance.^158^ It also signalled a shift in the disciplinary expertise guiding decision-making. In preparing response scenarios and corresponding budgets, planners were increasingly drawing on molecular data from a limited number of sentinel laboratories and mathematical predictions generated by disease modellers.^159^ The latter group had used mathematical models to aid the design of vaccination schedules and public health planning since the 1920s.^160^ However, the decades after 1990 saw a significant boost of modellers’ influence on the overall design of public health strategies as a result of revised epidemiological models, microbiology’s shift towards automated sero- and genotyping methods and the digitisation of reporting systems. In an age of budget cuts and securitised health thinking, modellers’ ability to transform the significant increase of available clinical and laboratory data into actionable risk scenarios was politically valuable. In conjunction with already prevalent cost-benefit assessments, necessarily hypothetical calculations of worst-case scenarios allowed decision-makers to justify the concentration of spending on a select number of threats.^161^

In the case of US laboratory infrastructures, ‘smart’ investment in a select number of specialist hubs and threats legitimised cuts to the more expensive traditional system of local public health laboratories with broad non-specialist testing remits. Hopes that new electronic networks, databases, and molecular typing technologies would be able to maintain effective disease surveillance despite growing gaps of baseline microbiological coverage underestimated how difficult this transition would be for many smaller laboratories. During the 1980s, pilots like the CDC’s 1984 Epidemiologic Surveillance Project or the WHO’s 1988 WHONet for AMR had highlighted the potential of information technology to standardise and accelerate clinical and laboratory reporting.^162^ However, the adoption of IT systems below the state level proved slow.^163^ By the time of the 1992 IoM report, all states were reporting electronic disease data to the CDC but several state laboratories still relied on paper forms. Even greater disparities in adopting electronic reporting occurred at the international level.^164^

In the absence of effective advocacy, the defunding of the US public health laboratory infrastructures was accelerated by the polarisation of health politics following the Clinton Administration’s 1993 attempt to establish universal health coverage.^165^ A 1994 report assessed the ‘crumbling foundation’^166^ of the US laboratory-based disease surveillance. According to a 1992 survey, ‘only skeletal staff exists in many state and local health departments to conduct surveillance for most infectious disease’.^167^ Out of 23 surveyed state laboratories, 22 reported hiring freezes or loss of positions.^168^ While planners focused on preparedness scenarios and Hollywood movies like *The Hot Zone* stoked public concerns about deadly ‘foreign’ pathogens like Ebola,^169^ the domestic public health laboratory infrastructure for the routine control of endemic disease was creaking. In the case of foodborne diseases, 12 states no longer had any dedicated personnel. In the case of HIV/AIDS, tuberculosis and childhood vaccine-preventable diseases, state data had become so unreliable that the CDC had to spend an additional $20 million per year for HIV/AIDS surveillance alone. Federal surveillance for multidrug-resistant tuberculosis was discontinued in 1986 before being reactivated in 1993 as a result of a significant incidence spike. With the exception of a few states like Washington, public health funding gaps were now the norm.^170^ In 1991, 78 towns with a combined population of 882,430 in Connecticut—the wealthiest US state—had no local access to full public health services, which often included a laboratory.^171^ According to CDC estimates, US state and PHS spending on public health amounted to ca. $14.4 billion in 1993 (ca. 1.6 per cent of total health care expenditure).^172^ Two years later, this figure was corrected downwards to about 1 per cent of total health care expenditure.^173^

Privatisation was another challenge. In contrast to the UK where commercial operators had been displaced by the NHS and PHLS,^174^ private sector testing had remained important in post-war America. Commercial laboratories provided testing for physicians, hospitals and local authorities.^175^ The CDC and APHL had tried to assure the quality of commercial testing and disease reporting with mixed results.^176^ Meanwhile, the growing political emphasis on fiscal restraint and management reforms created pressure on public health departments to outsource or commercialise further parts of their own laboratory services.^177^ In 1992, nearly half of surveyed state laboratory directors indicated that some form of privatisation of laboratory activities was under discussion.^178^ The commercial playing field was uneven. The ability of private laboratories to offer bulk testing for a select number of profitable diseases allowed them to undercut public laboratories, which also had to recoup the costs of preserving a broad skill reservoir for advanced testing of uncommon pathogens, surge capacity and other public health services.^179^ Many state laboratories already charged user fees, offered for profit assurance schemes for other laboratories, and accepted Medicare fees and third-party reimbursement for some services.^180^ However, payment systems proved difficult to establish and not all laboratories were allowed to keep generated revenues. In some cases, laboratory privatisation had to be reversed after it threatened core public health services as in the case of commercialised tuberculosis and sexually transmitted disease testing in New Jersey or of vendor instability in Pennsylvania. A 2000 review predicted that uncoordinated laboratory commercialisation and weakened surveillance could have dire public health consequences.^181^

The CDC continued to try to compensate for eroding local and state capabilities by improving the efficacy of existing laboratory networks and by investing in new molecular typing technologies and IT systems. In 1996, it established a network of electronically linked sentinel laboratories that could detect spikes of disease and employ PFGE molecular typing for *Escherichiacoli* and non-typhoidal *Salmonella* (PulseNet).^182^ Other federal programmes included the 1988 Public Health Laboratory Information System (PHLIS), the 1991 National Electronic Telecommunications System for Surveillance for the communication of weekly state reports on notifiable disease, the 1996 Tuberculosis Genotyping Network, and the 1996 National Antimicrobial Resistance Monitoring System (NARMS). Integration efforts were coordinated by the Health Information Surveillance System Board (est. 1995). In some states, the introduction of electronic systems reduced disease reporting from 35 days to 24 hours but this was sporadic and systems were often not interoperable.^183^ The USA also explored possibilities of strengthening international and hemispheric disease surveillance.^184^

Germany once again diverged from Anglo-American trends. In the 1980s politicians had also tried to contain rising health expenditure, streamline processes and introduce market elements in the form of ‘manacled competition’.^185^ Meanwhile, federal and state politicians faced growing pressure to upgrade public health arrangements in view of WHO priority health goals, the population-health oriented 1986 Ottawa Charter, and German reunification in 1990.^186^ However, Germany’s federal constitution and the highly decentralised nature of its public health system prevented both aggressive budget cuts and ambitious political shake-ups. In line with the country’s subsidiary principle (*Subsidiaritätsprinzip*), which stipulated that regulation by higher authorities should only occur where problems exceeded the abilities of lower ranking authorities, the result was a cautious series of public health reforms that simultaneously aimed to strengthen epidemic intelligence at the federal level, state-based epidemiological and laboratory competencies, and interstate collaboration. Although many components of East Germany’s health system were decentralised following reunification, parts of its high-performing microbiology infrastructure like the Institute for Experimental Epidemiology in Wernigerode were incorporated into the RKI. In Berlin, the status of health politics was upgraded with the separation of the Federal Ministry of Health (*Bundesgesundheitsministerium*) from the Federal Ministry of Youth, Family, Women and Health in 1991. Below the federal level, an increasing number of German states responded to national and international pressure by adopting statutory principles for public health and rationalising service provision—including laboratory services—at the communal level.^187^ As a result of interstate collaboration, long-standing recruitment problems were partially eased by no longer making public health an exclusively medical speciality and introducing postgraduate university courses.^188^

More ambitious reforms occurred after an international scandal shook the German infectious disease establishment. In 1987, West Germany had launched an Immediate Action Programme to Combat HIV/AIDS, which created ca. 700 specialist positions in public health offices and laboratories throughout the country. However, HIV/AIDS protection and testing infrastructures remained patchy. In 1993, the German public was shocked to learn that around 2,000 haemophiliacs and 600 transfusion recipients had contracted HIV due to contaminated blood and blood products.^189^ Although concerns about contaminated transfusions dated back to 1983, the *Bundesgesundheitsamt* and state officials had failed to halt the distribution of untested HIV-contaminated products by a company called UB Plasma until October 1993.^190^

The HIV scandal and concerns about multidrug resistant and emerging diseases prompted the largest shake-up of German infectious disease control since 1945. In 1994, the 118-year-old *Bundesgesundheitsamt* was dissolved. Its combined powers for drug regulation and communicable and non-communicable diseases were distributed to the Federal Institute for Drugs and Medical Devices (*Paul Ehrlich Institut*), the Federal Institute for Consumer Protection (*Bundesamt für Verbraucherschutz und Lebensmittelsicherheit*) and the RKI. The latter institution now also incorporated Germany’s AIDS Centre and Institute of Social Medicine and Epidemiology and thereby became Germany’s primary hub for communicable disease control.^191^ In response to calls for improved pathogen surveillance, the RKI established the first federal network of reference and consultant microbiology laboratories in 1995.^192^ Comprising 59 laboratories by 2015, the network spanned universities, federal and state laboratories, and private institutes. In keeping with German decentralisation, laboratories remained independent but received financial support and auditing from the RKI. In return, laboratories assumed responsibility for developing diagnostics, typing pathogens, maintaining reference strains, engaging in training and quality assurance for other laboratories, collaborating with international partners and detecting outbreaks. Integration of laboratory and clinical data streams was achieved by closely following the international template of epidemic intelligence and seconding a senior CDC epidemiologist from Atlanta to Berlin to advise German authorities. Starting in 1996, the RKI used the new stream of disease data to issue a weekly epidemiological bulletin, formed a Committee for Infectious Disease Epidemiology, updated nosocomial infection guidelines, and established an EIS-modelled field epidemiology training course.^193^ In 1998, it published the first German Health Survey.^194^ In the same year, new EU guidelines also triggered inter-party drafting of an ambitious reordering of Germany’s infectious disease legislation and plans for integrated AMR monitoring (see Part Four of this article).^195^

After four decades of relative neglect, the combined effects of reunification, scandals, and EU integration made decision-makers in Berlin firmly commit to strengthening and integrating Germany’s decentralised hinterland of ca. 360 state and communal *Gesundheitsämter*.^196^ However, in contrast to the USA and the UK, boosting epidemic intelligence did not come at the cost of cuts to local public health laboratory services. Bound by the subsidiary principle, reformers resorted to the traditional strategy of overcoming resistance to centralisation by simultaneously investing in state-based systems. With public health and prevention expenditure making up a stable percentage of rising total health expenditure, the result was more money for the entire public health system.^197^

## Part Four: The Age of Preparedness (ca. 2000–2020)

Concerns about the functionality of public health and laboratory systems acquired new urgency as a result of the 2001 anthrax attacks and the 2003 SARS-CoV pandemic. Large-scale reforms occurred in each analysed country. Injections of public money, public health reforms and new genomic technologies dramatically boosted authorities’ ability to quickly identify and react to emerging outbreaks. On both sides of the Atlantic, planners continued to hope that targeted high-tech surveillance in specialised laboratory hubs and integrated epidemic intelligence would be able to stop outbreaks in their tracks. What would happen if this line of defence failed was the subject of numerous outbreak movies like *Contagion* (2011) but failed to refocus decision-making on the robustness of underlying public health laboratory infrastructures.

In Britain, the decade between 2003 and 2013 saw the biggest shake-ups of infectious disease surveillance since the creation of the EPHLS in 1939. The first shake-up was the result of the Blair Administration’s attempt to reform British health, social care and consumer protection whilst also devolving powers to the national governments of Wales, Northern Ireland and Scotland. In the wake of the 1996 mad cow disease (BSE) crisis, a new Food Standards Agency (est. 2000) was placed in charge of monitoring the food chain. One year later, the Department for Environment, Food and Rural Affairs assumed responsibility for agriculture and environmental health and the creation of 303 NHS Primary Care Trusts (PCTs) led to a significant restructuring of community-level public health. Taking over from the 95 now dissolved district health authorities, PCTs were tasked with providing and commissioning primary, community and secondary health services. Preserving integrated infection control abilities did not rank high on reformists’ agenda. Instead, the creation of PCTs fragmented the post-1988 realignment of local and district-level epidemiology, microbiology, clinical, and local authority services as well as the existing public health workforce of CCDCs and DPHs.^198^

The PHLS did not escape reformism either. In 2003, the now 64-year-old organisation was disbanded and merged into a new Health Protection Agency (HPA). The HPA was the brainchild of CMO Liam Donaldson and his 2002 *Getting Ahead of the Curve* report.^199^ Referring to the 2001 US anthrax attacks, Donaldson’s report mirrored the preparedness language of the 1992 IoM report by painting a dark picture of infectious disease, bioterrorism, and chemical and nuclear threats. It also stated that a lack of integrated intelligence and response capabilities undermined national security. In the case of infectious disease, the report acknowledged the successful PHLS response to the limited number of British SARS cases in 2003 but called for an integration of all aspects of health protection (infectious disease, chemical and radiation hazards) under one organisational roof. This would entail major changes to Britain’s public health laboratory infrastructure.^200^

In a radical move, *Getting Ahead of the Curve* recommended ending the traditional separation of local public health and clinical microbiology and concentrating public health resources on the centralised provision of specialist services. Control over all local PHLS laboratories should be transferred to the NHS—thereby essentially implementing the 1985 DHSS recommendations and creating a CDC-like agency with one centre and a few regional hubs. Rather than controlling public health microbiology at the local level, the HPA would act as a coordinating centre of epidemic intelligence and provide microbiological reference, consulting and rapid response services to the NHS, British government and local authorities. It was envisioned that HPA experts would closely coordinate with NHS Pathology Services in England, who would now provide all local microbiology services and report relevant public health data to the HPA. Should it be required, additional NHS testing could be commissioned. Reformers hoped that transferring the distinct network of PHLS laboratories to the NHS would rationalise local microbiology services and improve diagnostic, assurance, and reporting standards via centralised NHS oversight. They also hoped that NHS PCTs, Strategic Health Authorities and laboratories would not only support the HPA during outbreaks but also devote sufficient resources towards maintaining routine public health microbiology outside of emergencies.^201^ These expectations proved difficult to meet.

The HPA was formally established on 1 April 2003 with four bases at Colindale, Porton Down,^202^ Chilton, and South Mimms. It comprised a health protection services division, which would integrate a laboratory and a new national epidemiology service, a microbiology services division, a centre for radiation, chemical and environmental hazards, and a division for biological standards and control.^203^ The HPA’s Microbiology Network comprised only 9 laboratories (of which two were located in London) while the former PHLS’ 38 local laboratories were transferred to the NHS (Figure 2). To facilitate this transfer without compromising local public health laboratory services, the government guaranteed a stable budget until April 2005 after which money should come from the NHS or direct HPA commissioning.^204^ Similar to the CDC’s EIS, specialist Health Protection Units (HPUs) were supposed to act as the ‘eyes and ears’ of the HPA, liaise with local and NHS authorities, respond to health protection incidents and keep ‘a finger on the health protection pulse locally’.^205^

The creation of the HPA was greeted with mixed feelings by PHLS staff.^206^ Giving evidence to the House of Lords, officials repeated Howie’s 1985 warnings about trading off local laboratory coverage against centralised epidemic intelligence and lacking NHS incentives to adequately fund public health microbiology:

There is real and general concern about the capacity to deliver enhanced surveillance or to mount an acute response. (…). There appear to be no plans as yet for providing any material incentive for NHS laboratories to rise to the public health challenge, and a fear that in local tussles about funding, money will be diverted from public health purposes.^207^

Similar to problems within the CDC, it was also unclear whether HPA management would neglect infectious disease over chemical, radioactive or chronic disease threats.^208^ Reflecting on the PHLS-led response to the 2003 SARS pandemic,^209^ virologist Philip Mortimer warned that the UK had been lucky because of the nature of the pathogen and England’s integrated public health laboratory infrastructure. Mortimer noted that over relying on mathematical disease modelling and epidemic intelligence in the absence of sufficient local laboratory capacity, contact tracers and isolation beds could prove costly during future pandemics. What was needed was broad infrastructural investment:

… it should not be assumed that a recurrence of SARS is therefore unlikely, or that a further outbreak would be controllable. (…). If there are weaknesses or deficiencies it should not be thought that they can or should be repaired by quick fixes each time an acute threat materialises. Such expenditures fail to build the infrastructure needed to maintain a comprehensive capacity for rapid and technologically appropriate response to new pathogens, and over time they distort facilities and so hinder the effective management of the laboratory.^210^

Mortimer’s warnings proved prescient. Later remembered as a ‘painful birth’,^211^ the first years of the HPA were characterised by a slow merging of once independent institutions. In contrast to Donaldson’s vision of a ‘customer-focussed’ integrated public health service,^212^ relations within the HPA were described as ‘territorial’, which ‘led to a lot of wasted energy on positioning small politics and defending them’.^213^ Coordinating HPA and NHS services was equally challenging. In the case of infectious disease control, recruitment problems and NHS underinvestment in microbiology services meant that building effective collaborations between regional HPA hubs and local laboratories and authorities was challenging.^214^ Within a year of the end of ring-fenced funding, a 2006 review of NHS Pathology Services in England warned that it was not obvious what kind of accountability NHS Trusts had for financing local public health microbiology services in the medium- to long term.^215^ Stripped of its network of local laboratories, Britain’s flagship public health hub in Colindale was at risk of becoming a giant on clay feet.

The effects of losing local public health laboratory services were compounded by confused official responsibilities for infection control. PCTs were charged with protecting community public health but relied on professionals in the HPA’s headquarters and HPUs for response management. Local authorities were responsible for protecting the public from infectious disease and appointing a CCDC. However, CCDCs were no longer responsible to the NHS DPH but were employed by the HPA as part of its HPUs. Meanwhile, the HPA was dependent on PCTs, NHS England Pathology Services and local authorities for routine clinical and microbiological data generation and for mounting outbreak responses.^216^ At the national level, the HPA’s post-2005 status as a non-departmental authority also risked creating parallel hierarchies with the NHS and Department of Health during public health emergencies.^217^

The 2008 financial crash exacerbated problems with severe funding cuts for the HPA. Between 2009 and 2013, HPA staffing decreased from 4,100 to 3,700 (9.7 per cent). Meanwhile, the agency’s core budget declined by over 26 per cent from £193 million (2009/2010) to £142 million (2012/2013). In response, the HPA became increasingly dependent on extramural grants and private funding for special programmes and research. Similar to the USA (see below) and parallel developments in international health,^218^ relying on rigid earmarked rather than stable budgets could distort management priorities towards targeting ‘profitable’ funder priorities while exacerbating funding shortages for routine public health services.^219^

In 2010, the new Conservative government launched a policy of fiscal austerity. Despite successful HPA responses to the 2006 polonium killings and 2009 H1N1 pandemic,^220^ a further economic slimming and centralisation of public health was decided in 2011. In 2013, the HPA and its eight regional laboratories were integrated into Public Health England (PHE). The goal was to create a mammoth public health service, which would merge over 5,000 staff from 129 organisations. In contrast to the relative political autonomy of PHLS and HPA decision makers, PHE was an executive agency under direct control of the Department of Health and would be managed by a chief executive officer with no formal training in medicine or public health.^221^ Although PHE was supposed to be free to speak on issues relating to the nation’s health,^222^ the extent of this freedom and the ability to act autonomously in the name of public health was unclear.

The creation of PHE was part of the second shake-up of British health care within a decade. The 2012 Health and Social Care Act abolished New Labour’s PCTs and stripped the NHS of its responsibility for local public health services, which were returned to local authorities. According to the new doctrine of democratic localism,^223^ upper tier (county councils) and unitary (metropolitan) local authorities in England would establish multiparty Health and Wellbeing Boards (HWBs), draw on expertise from lower district levels (e.g. in environmental health), and appoint a DPH for the combined delivery of public health, health, and social services at the local level. Select primary care services such as screening, immunisation, and public health services for children and pregnancy remained part of the NHS and were coordinated by Clinical Commissioning Groups and NHS England.^224^ Similar to the HPA, PHE would act as an epidemic intelligence centre providing specialist reference, global health, and cost-effectiveness services while supporting local HWBs and DPHs with health protection teams. PHE’s coordinating role was further boosted by the 2010 Health Protection (Notification) Regulations, which updated disease and reporting requirements and gave local authorities more flexible powers to deal with emergencies.^225^

(Re)-implementing effective localism was undermined by ongoing budget cuts. To maintain outbreak preparedness and routine public health services, PHE relied on support from the NHS and local authorities. This included sufficient funding for local public health laboratory services and reporting by NHS Pathology Services as well as the ability and willingness of local authorities and NHS managers to adhere to formal public health outcome frameworks.^226^ Shrinking NHS and council budgets made this collaboration challenging. Ministers had initially promised to ring-fence the public health budget for local authorities until 2016. However, an in-year cut of £200 million in 2015 was followed by a further 9.6 per cent cut over the next 5 years. This amounted to a real term reduction of public health spending from £3.5 billion in 2015–16 to just over £3 billion in 2020–21 (a reduction from ca. 4.1 per cent to 2.5 per cent of total health expenditure).^227^ PHE itself had to absorb a 30 per cent funding cut between 2013 and 2017. Severe local funding shortages were exacerbated by ‘top slicing’ whereby councils reallocated ring-fenced public health budgets to other local services impacting health and wellbeing.^228^ Meanwhile, retirements and recruitment problems left 17 per cent of DPH posts vacant in 2017.^229^

Ahead of the biggest pandemic event since 1918, the once formidable public health laboratory infrastructure in England was smaller than at any point since 1939. Despite access to cutting-edge molecular typing, world-renowned experts, and integrated electronic reporting, the UK’s centre of epidemic intelligence in Colindale had reduced political autonomy to act in the name of public health, less contact with local microbiological and public health services, and little surge testing capacity to fall back on should its national and regional reference laboratories be overwhelmed.

The growing centre–hinterland divide and discrepancy between preparedness rhetoric and actual laboratory capacity were mirrored in the USA. Between 1999 and 2020, a brief effervescence of public health funding and reform was followed by a prolonged phase of budget and personnel problems. In 1999, a review by the non-partisan Government Accountability Office (GAO) reported ongoing gaps in the US laboratory surveillance and disease reporting. Almost half a century after their creation, it remained voluntary for states to adopt the annually reviewed list of notifiable diseases issued by the CDC and Council of State and Territorial Epidemiologists. While this arrangement enabled states to opt out of testing for diseases that were rare in their territory and focus resources on a national 'core list' of reportable diseases, it also created surveillance gaps for emerging threats like hepatitis C and AMR.^230^ Technology differences exacerbated surveillance gaps. In 1999, slightly more than half of state laboratories conducted molecular PFGE-typing. However, genotypic data did not necessarily correlate with phenotypic data produced by non-molecular surveillance in other states. Similar problems characterised the adoption of IT systems. Seven years after the 1992 IoM report, 40 per cent of state laboratories did not or barely used computerised systems to receive surveillance data and 21 per cent barely made use of computerised systems to transmit data.^231^ Concerns were also raised about the ongoing commercialisation of public testing and patchy disease reporting by out-of-state laboratories.^232^ Worryingly, over 30 state laboratory directors warned that lucrative fee-based genetic screening and tests for regulatory and licensure programmes were skewing routine laboratory operations ‘away from testing services beneficial to the entire community [like influenza surveillance]…’.^233^

Powerless to override state decision-making, the CDC tried to mobilise financial resources and increase the efficiency of existing public health laboratory networks by stressing biosecurity scenarios. A 1999 CDC report declared that Americans were ‘a Nation at risk’.^234^ The USA was unprepared for new threats posed by the rapid dissemination of disease via global travel, AMR, and bioterrorism. Preparedness could, however, be improved by investing in a new electronic CDC Health Alert Network and by setting out regularly reviewed state improvement plans for public health. Essentially repeating recommendations from the 1988 and 1992 IoM reports, the CDC announced that it would support states with extramural grants, training, technical assistance, and evaluations. Further recommendations centred on updating public health education and creating integrated databanks. In response to the GAO, the need to improve laboratory capabilities in all federal and state health departments was also mentioned.^235^ The laboratory capabilities singled out for improvement were defined by contemporary preparedness concerns. In 2000, the CDC announced the creation of a rapid laboratory response network for biological and chemical agents with integrated surveillance and a strategic plan for bioterrorism by 2004.^236^

Outside of Atlanta, the APHL tried to use Congress’ Healthy People 2010 initiative to promote a broader vision of public health laboratory capacities. Although it also referenced bioterrorism threats, its 2000 consensus vote on core functions of state public health laboratories only listed emergency response capabilities on place 8 of 11 – below the provision of diagnostic, surveillance, reference, quality assurance, and data management services.^237^

The 2001 World Trade Centre and anthrax attacks created further momentum for the enactment of preparedness-oriented reforms of the US public health laboratory infrastructures. According to the IoM, 2001 had revealed:

… vulnerable and outdated health information systems and technologies, an insufficient and inadequately trained public health workforce, antiquated laboratory capacity, a lack of real-time surveillance and epidemiological systems, ineffective and fragmented communications networks, incomplete domestic preparedness and emergency response capabilities, and communities without access to essential public health services.^238^

During fiscal year 2002, the Bush Administration increased already rising federal preparedness spending. Of the $2.9 billion bioterrorism appropriations bill signed on 10 January 2002, $1.1 billion were dedicated to upgrading bioterrorism preparedness, infectious-disease surveillance, network integration, and hospital surge capacity.^239^ The CDC and APHL responded by establishing a dedicated Laboratory System Improvement Program (L-SIP). Regulators also developed a Model State Emergency Health Powers Act that could be implemented by states to better integrate interstate emergency capabilities.^240^ At the state and local level, post-9/11 funding led to a brief surge of laboratory spending. Many of the ca. 2,000 public health laboratories upgraded equipment, invested in 24/7 availability and IT networks, hired staff, and began offering molecular typing services.^241^ At the federal level, the CDC expanded its epidemic intelligence capabilities by promoting existing surveillance systems like NARMS, VetNet for *Salmonella* in animals, FoodNet for the surveillance of foodborne diseases, and OutbreakNet for PFGE data sharing.^242^ Ideals of real-time surveillance also led to the creation of new laboratory networks. The CDC officials collaborated with the FDA and the new Department of Homeland Security to create the Food Emergency Response Network (FERN, 2004) and Environmental Public Health Tracking Network (EPHT, 2005).^243^ In 2003, the US public health authorities used their integrated resources to mount an effective response to SARS^244^—although the CDC subsequently highlighted the need for additional local testing capacity.^245^ The Pandemic and All-Hazards Preparedness Act (PAHPA) and the 2006 creation of the Biomedical Advanced Research and Development Authority (BARDA) further enhanced authorities’ ability to prepare for projected emergencies with strategic stockpiles and research.^246^

Whether the surge of preparedness funding, which peaked in 2004, was sufficient to shore up baseline US public health capacity was debatable.^247^ The new money was spent unevenly and biosecurity priorities did not necessarily align with broader infection control needs. At the federal level, Homeland Security and CDC officials repeatedly clashed over how much funds should be earmarked for bioterrorism threats like smallpox and anthrax and who should have control over new surveillance data.^248^ Although states and counties used federal preparedness funds to upgrade laboratories, IT systems, and typing technologies, some complained that federal bioterrorism priorities were draining resources from routine laboratory surveillance for other pathogens.^249^ The relative neglect of day-to-day laboratory testing in favour of a select number of high-priority targets was exacerbated by the Bush Administration’s decision to slash the federal budget for preventing chronic disease, promoting health, and controlling infectious disease by $67 million and impose flat spending on other programmes.^250^ Despite parallel preparedness money, state-level fiscal problems and increased Medicaid and Medicare expenditure resulted in public health budget declines in more than half of the US states between 2003 and 2004.^251^ According to a 2007 survey of state public health laboratories, 64 per cent had insufficient staff levels, many could not afford regular training, and 43 per cent remained unable to mandate the submission of isolates and specimens from commercial laboratories.^252^

Despite these problems, the USA mounted an effective response to the 2009 H1N1 pandemic. New molecular capabilities meant that it took only 2 weeks from the identification of the novel virus to the creation, emergency FDA authorisation, manufacture, and distribution of a rapid RT-PCR-based test. Rapid FDA licensing had in part been possible because of prior CDC and BARDA investment in a platform technology (five target assay) for rapid influenza testing that could be quickly modified. In line with its preparedness protocols, the USA had also stockpiled reagents.^253^ Within a few days of modifying the test, representatives from 44 public health laboratories had visited the CDC for training. Although false negatives resulting from less accurate rapid tests caused problems, PCR-based testing, electronic reporting, and vaccine development bolstered confidence in the US pandemic preparedness and the ability of integrated networks of public and private laboratories to overcome chronic laboratory capacity problems in emergency situations.^254^ Looking back at the H1N1 response, the APHL highlighted the importance of preparedness research, hybrid public–private lab networks, and genomic technologies for future pandemics:

Before [H1N1], the federal government would have to make a lot of tests, and we’d send them out, and people would use them … With people being able to do their own PCR, we could put up the recipe and let people make that themselves. It’s a much more robust approach for people able to ramp up testing for a novel pathogen.^255^

The fact that non-governmental academic, private and clinical laboratories could validate ‘homebrew’ tests against a published gold standard had stopped public health laboratories from being overwhelmed by a flood of specimens.^256^ In the midst of resulting optimism about new technologies and hybrid networks, it was easy to forget that future crises might involve less well-known pathogens and that state public health laboratories had played a key role in boosting testing, providing quality assurance, and coordinating responses outside Atlanta.^257^

The resilience of this public health laboratory infrastructure faced accelerating threats following the 2008 financial crash. With the US states experiencing severe fiscal pressure, public health laboratories were forced to reduce or commercialise testing services. Federal decision-makers did little to counteract these cuts. In 2009, a large part of the Obama Administration’s $5.8 billion of H1N1 surge funding was spent on vaccination and only a small percentage of funds reached laboratories.^258^ Between 2008 and 2010, over 44,000 jobs were lost in state and local health departments including public health physicians, laboratory specialists and epidemiologists.^259^ Staff shortages not only increased pandemic vulnerabilities but also compromised routine infectious disease surveillance. In 2010, 24 per cent of the US states could not submit 90 per cent of *E. coli* molecular typing results to CDC’s PulseNet database within four working days.^260^ Despite their importance during the 2009 pandemic, porous laboratory accreditation frameworks impeded quality assurance and data sharing between private and public laboratories as well as between local, state and federal agencies.^261^

Challenges for public health laboratories increased following the enactment of the Obama Administration’s 2010 Affordable Health Care Act. Medicare and Medicaid had been significant revenue sources for public health laboratories. However, the vast increase of people eligible for basic testing raised fears about the future of direct public financing, necessitated a significant upgrade of public billing systems, and increased competition with commercial laboratories—with state laboratories still required to maintain core functions and provide services to uninsured Americans.^262^ The 2012 decision to reallocate $6.25 billion of the originally promised $15 billion boost to public health and subsequent sequestrations further increased problems for public health laboratories. After briefly rising to ca. 3.18 per cent of total health spending in 2002, the US expenditure on public health declined to ca. 2.65 per cent of total health spending in 2014 with a further fall to 2.4 per cent predicted by 2023.^263^ Although CDC planners resorted to the familiar approach of trying to compensate for cuts with improved IT and next generation sequencing,^264^ recruitment problems and budget cuts—including President Trump’s announcement on 10 February 2020 of a 16 per cent reduction of CDC funding—severely undermined the resilience of the US public health laboratory infrastructure just before it would be tested to its limit.^265^

Of the three analysed countries, Germany was the only one to mostly strengthen laboratory services between 2000 and 2020. Driven by EU requirements and demands for integrated European surveillance networks, a new infectious disease law (*Infektionsschutzgesetz*) came into force in 2001. In line with previous reforms, key public health competences remained in the hand of state and communal authorities, but the 2001 law enhanced authorities’ ability to respond to outbreaks with improved vaccination, surveillance and police powers—including the right to impose quarantine and job bans. The federal centre in Berlin was empowered to impose federal control in the case of disease events of national significance. State-level and federal disease reporting requirements were unified and upgraded with Berlin now able to demand laboratory and disease surveillance data that included personal information during emergencies.^266^

As the federal epidemiological centre, the RKI focused more resources on curbing nosocomial infections, increasing vaccine coverage (by incorporating Germany’s permanent vaccine commission), and improving infectious disease surveillance.^267^ In doing so, the RKI continued to build on the CDC model of epidemic intelligence. Sentinel and reference laboratories were strengthened. Doctors, hospitals, and private laboratories were required to report both suspected and confirmed reports of notifiable diseases to communal or state health authorities, who would then communicate routine information and test results to the RKI in an anonymised form.^268^ To facilitate data integration, the RKI emulated the USA and the UK investment in IT systems by rolling out SurvNet@RKI as the first integrated German electronic reporting platform for infectious disease among civilian authorities on 1 January 2001—and in the armed forces in 2006.^269^ Preparedness planning featured alongside overall systems’ strengthening. Following WHO guidance from 1999, Germany developed its first pandemic influenza plan—well after similar plans in the USA and the UK—in 2001. Reporting systems were updated after the 2011 enterohemorrhagic *E. coli* (EHEC) epidemic exposed ongoing communication gaps between different states. A new electronic notification system (*Deutsches Elektronisches Melde- und Informationssystem für den Infektionsschutz*) was trialled between 2016 and 2020.^270^

Repeatedly upgraded after 2001, Germany’s new infectious disease legislation addressed long-standing organisational constraints at the communal and state level whilst also integrating national networks and strengthening federal oversight. This did not mean that variations in state public health arrangements ceased or that the RKI in Berlin became dominant. A further attempt to centralise and rationalise regulations and competencies was rejected by states in 2005.^271^ At the political level, the 2001 reform instead solved long-standing problems of fragmented federal, state and communal responsibilities and the relative neglect of infectious disease control. Following the subsidiary principle, reforms at the top did not necessarily lead to cuts at the bottom. While English reforms fragmented and reduced local infection control capabilities and the US surge funding prioritised preparedness over routine laboratory services, German reforms strengthened both emergency and routine services. Between 2000 and 2015, state, federal, and health insurance spending on prevention and public health services amounted to between 3.27–3.52 per cent of rising total health expenditure. Money was spent at the federal, state and communal levels.^272^ The more complicated nature and looser integration of German infection control and laboratory infrastructures meant that the RKI’s epidemic intelligence capabilities could not match those of the CDC or PHE. However, German authorities could draw on a public health hinterland with sufficient power, autonomy and coordination to react flexibly should centres of excellence be overwhelmed. Despite coordination problems,^273^ the hybrid nature of German systems building—coupled with the early decision to incentivise and incorporate testing by commercial laboratories—allowed for a rapid 2020 surge of testing and access to resulting data by local authorities.^274^

## Conclusion: Upgrading the Hinterland

On the eve of COVID-19, assessing the preparedness of different public health infrastructures was thus deceptive: Anglo-American centres of excellence wielded significant international influence, had developed sophisticated molecular typing and electronic notification systems for existing laboratory assets, and had fine-tuned a model of epidemic intelligence that could rapidly deploy experts and stockpiled equipment to quell localised outbreaks. Despite ongoing cuts and reforms, responses to the 2003 SARS and 2009 H1N1 outbreaks engendered confidence that Anglo-American public health systems could protect citizens from new or familiar pathogens.

However, beyond the centre, the wider state of public health laboratory infrastructures was worrying. Since the late 1970s, decades of budget reductions and attempts to streamline laboratory services had eroded local testing and microbiological expertise. Routine laboratory services for many infectious diseases had been cut or outsourced and staff shortages were significant. Should centres of epidemic intelligence fail to stamp out an emerging epidemic or pandemic event, there was little hinterland capacity to fall back on to protect the public’s health.

These infrastructural weaknesses had been highlighted by multiple reports and exercises like the UK’s 2016 Operation Cygnus.^275^ Interestingly, they were, however, not adequately taken account of by international preparedness reviews. Conducted by experts, who either originated from or had been trained in the USA or the UK, prominent reviews followed the narrow priority setting approach of 1990s pandemic planning by concentrating on key performance indicators including detection, reporting, and response capabilities for specific pathogens as well as health systems’ capacity to treat sick workers.^276^ Missing from this mode of analysis was a focus on the non-specialist public health laboratory services, whose surge capabilities, quality assurance, and expertise in collaborating with other local public health services connected national nerve centres of epidemic intelligence to the rest of the country. The relative political autonomy of public health centres to act swiftly was also not taken account of. The result was a mode of review that was designed to confirm rather than challenge orthodoxies of centralised intelligence and lean surveillance and consistently ranked countries with more decentralised resources lower than those with individual hubs of excellence.^277^

While hindsight is cheap and history provides no simple lessons, focusing on the long-term development of the laboratory infrastructures underpinning public health systems can provide insights for planning going forward. One of these insights is the importance of context and time when it comes to creating and maintaining resilience. As this article has shown, laboratory infrastructures evolved to fit the specific needs, political systems, and infection control visions of their respective national contexts. While the concerns shaping this evolution were often similar, the degree of central control, integration, and efficiency of resulting infrastructures differed: Britain's PHLS evolved its laboratory network to fit around an already existing system of local epidemiology and public health consisting of MoHs and local authorities; the CDC’s concept of epidemic intelligence was developed in response to concerns about biological attack and the need to coordinate autonomous state public health laboratory systems; West Germany’s laboratory system was shaped by existing communal traditions and by the post-war focus on de-Nazifying and decentralising public health.

The specificity of each laboratory infrastructure meant that reforming it required careful deliberation of how it fit into the wider public health ecosystem as well as sufficient time and fiscal resources to ensure that vital components could adapt to new challenges and visions of infectious disease control. Both resources were usually in short supply. With attention for infectious disease control declining and post-1960s planners under pressure to cut costs, the CDC template of epidemic intelligence proved an attractive export. The mix of centralised epidemiological oversight, integrated reporting networks, and rapid response capabilities offered significant strategic and cost advantages when it came to moving surveillance from the individual to the population level and targeting a select number of diseases. What was, however, increasingly forgotten was that this ‘lean’ template of epidemic intelligence had only become possible as a result of post-1920s investment in local and state laboratory infrastructures that were capable of supplying large volumes of microbiological data and which were now being pruned in the name of cost-effectiveness. In both the USA and the UK, planners and politicians hoped that technological advances like electronic reporting, automated processing, and—later—molecular typing would compensate for reduced local services. Once established as a default reform pathway, visions of cost-effective epidemic intelligence, real-time surveillance, and preparedness for select biosecurity scenarios overshadowed warnings about gaps in routine public health laboratory services. Funding surges in response to crises like 9/11 were too short-lived and focused on individual pathogens to compensate for these growing infrastructural weaknesses. The German case study is the exception that proves the point. It was the difficulty of pushing bigger reform packages through Germany’s compromise-oriented federal system that gradually created a relatively stable balance between centralised epidemic intelligence and ongoing support for autonomous state and local laboratory networks.

Paying close attention to the described case studies is to appreciate the potential of epidemic intelligence in pooling and analysing epidemiological information, the natural limits of specialist hubs when it comes to the provision of routine public health services, and the dangers of one-size-fits-all thinking in international health. Going forward, it means paying greater political and academic attention to the mundane technical infrastructures—like laboratories—that underpin public health both during and outside of outbreaks. Focusing on these mundane infrastructures complicates media narratives foregrounding the role of centralised pandemic decision-making in Atlanta, Colindale, and Berlin. It also provides an important counterpoint to a popular genre of science writing that routinely emphasises the work of lone ‘disease detectives’ and elite scientists within infection control.^278^ And finally, it challenges intellectual path dependencies that see even more investment in preparedness, biosecurity, and specialist epidemic intelligence hubs as the only answer to the current pandemic.^279^

## Footnotes

1 ‘WHO Director General’s Opening Remarks at the Media Briefing on COVID-19 - 16 March 2020’, World Health Organisation, 2020.

2
*Global Health Security Index. Building Collective Action and Accountability* (Baltimore: NTI/Johns Hopkins Bloomberg School of Public Health, 2019); C. Tallack and H. Krelle, ‘Comparing G7 Countries: Are Excess Deaths an Objective Measure of Pandemic Performance?’, *The Health Foundation*, 10 June 2021. Available from: https://health.org.uk/publications/long-reads/comparing-g7-countries-are-excess-deaths-an-objective-measure-of-pandemic-performance (accessed 26 April 2022); 'Germany's 'bottom-up' testing keeps coronavirus at bay', Financial Times, 18 October 2020.

3 For some examples, see: E. Yong, ‘We’re Already Barrelling toward the Next Pandemic’, *Atlantic*, 29 September 2021; F. Lawrence *et al.*, ‘How a Decade of Privatisation and Cuts Exposed England to Coronavirus’, *Guardian*, 31 May 2020; P. Blackburn, ‘Austerity—Covid’s Little Helper’, *British Medical Association*, 08 October 2020. Available from: https://www.bma.org.uk/news-and-opinion/austerity-covid-s-little-helper (accessed 26 April 2022); J. Baumann, ‘Decades of Yo-Yo Funding Hampering Covid-19 Response’, *Bloomberg Law*, 09 June 2020; journalists like Laurie Garrett have warned about funding cuts since 2000; L. Garrett, *Betrayal of Trust: The Collapse of Global Public Health* (New York: Hyperion 2000).

4 P. Clark *et al*., ‘How the UK Got Coronavirus Testing Wrong’, *Financial Times*, 27 March 2020; F. Fukuyama, ‘The Pandemic and Political Order. It Takes a State’, *Foreign Affairs*, 2020, 99, 26; S. Crosby *et al.*, ‘All Bets Are Off for Measuring Pandemic Preparedness’, *Think Global Health*, 2020, 30 June 2020; E. Lipton *et al.*, ‘The CDC Waited “Its Entire Existence for This Moment.” What Went Wrong?’, *New York Times*, 03 June 2020; T. Helm, E. Graham-Harrison, and R. McKie, ‘How Did Britain Get its Coronavirus Response So Wrong?’, *Guardian*, 19 April 2020; ‘Domic Cummings: Thousands died needlessly after Covid mistakes’, *BBC News*, 26 May 2021.

5 J. A. Patel *et al*., ‘Poverty, Inequality and COVID-19: The Forgotten Vulnerable’, *Public**Health*, 2020, 183, 110; C. Dodds and I. Fakoya, 'COVID-19: ensuring equality of access to testing for ethnic minorities', *BMJ*, 2020, 369, m2122; A. L. Best *et al.*, ‘Institutional Distrust among African Americans and Building Trustworthiness in the COVID-19 Response: Implications for Ethical Public Health Practice’, *Journal of Health Care for the**Poor**and**Underserved*, (2021), 32, 90; L. Jones and S. Hameiri, ‘COVID-19 and the Failure of the Neoliberal Regulatory State’, *Review of International Political Economy*, 2021, 1–25.

6 See, for example, contributions to the 2020 Centaurus spotlight issue on ‘Histories of Epidemics in the Time of COVID-19’, *Centaurus*, May (2010), 219–380; J.-P. Gaudillière and C. Beaudevin, ‘Covid-19 and Global Health, Seen from France: The End of a “Great Divide?’, *Somatosphère: Science, Medicine, and Anthropology*, 2020; A. Saltelli *et al.* ‘Five Ways to Ensure that Models Serve Society: A Manifesto’, *Nature*, 2020, 482–84.

7 S. L. Star and K. Ruhleder, ‘Steps toward an Ecology of Infrastructure: Design and Access for Large Information Spaces’, *Information Systems Research*, 1996, 7, 111–34.

8 A. Cunningham and P. Williams (eds), *The Laboratory Revolution**in**Medicine* (Cambridge: Cambridge University Press, 2002); A. Hardy, *Salmonella Infections, Networks**of**Knowledge*, *and**Public Health in Britain, 1880–**1975* (Oxford: Oxford University Press, 2015); R. Wall, *Bacteria in Britain, 1880–1939* (Abingdon: Routledge, 2015); P. M. H. Mazumdar, ‘“In the Silence of the Laboratory”: The League of Nations Standardizes Syphilis Tests’, *Social History of Medicine*, 2003, 16, 437–59.

9 L. Engelmann, ‘A Box, a Trough and Marbles: How the Reed-Frost Epidemic Theory Shaped Epidemiological Reasoning in the 20th Century’, *History and Philosophy of the Life Sciences*, 2021, 43, 1–24; G. Milward, *Vaccinating Britain: Mass Vaccination and the Public Since the Second World War* (Manchester: Manchester University Press, 2019).

10 With a few exceptions, this area of inquiry has been left to anthropologists and STS scholars, see for example: C. Beaudevin *et al.*, ‘“Test, Test, Test!”: Scarcity, Tinkering, and Testing Policy Early in the COVID-19 Epidemic in France’, *Medicine Anthropology Theory*, 2021, 8, 1–31; N. Fortané and F. Keck, ‘How Biosecurity Reframes Animal Surveillance’, *Revue d'anthropologie des connaissances*, 2015, 9, a-l; F. Vagneron, ‘Surveiller et s’ unir? Le rôle de l’OMS dans les premières mobilisations internationales autour d’un réservoir animal de la grippe’, *Revue d’anthropologie des connaissances*, 2015, 9, 139–162; A. Hardy, 'Salad Days. The Science and Medicine of Bad Greens, 1870-2000', in A. Creager and J.-P. Gaudillière (eds.), *Risk on the Table: Food Production, Health, and the Environment* (New York: Berghahn, 2021), 29-54.

11 P. Chakrabarti, *Bacteriology in British India: Laboratory Medicine and the Tropics* (Rochester: University of Rochester Press, 2012); J. T. Crane, *Scrambling for Africa: AIDS, Expertise, and the Rise**of**American Global Health Science* (Ithaca: Cornell University Press, 2013); C. I. R. Chandler, ‘Current Accounts of Antimicrobial Resistance: Stabilisation, Individualisation and Antibiotics as Infrastructure’, *Palgrave**Communications*, 2019, 5, 1–13; C. Caduff, *The Pandemic Perhaps* (Berkeley: University of California Press, 2015); A. Lakoff, *Unprepared: Global Health In A Time**of**Emergency* (Berkeley: University of California Press, 2017), 67–117; W. Koster *et al.*, ‘An Oral History of Medical Laboratory Development in Francophone West African Countries’, *African Journal**of**Laboratory Medicine*, 2021, 10, 1–10.

12 A recent volume on the history of Global Health is indicative of this trend by making important contributions to mapping the ambivalent impact of biomedical interventions in the Global South but mostly glossing over the functioning of the same interventions in the Global North, J.-P. Gaudillière *et al.* (eds), *Global Health and the New World Order* (Manchester: Manchester University Press, 2020).

13 Hardy, *Salmonella Infections*, 23–42; M. Harrison, *Disease and the Modern World. 1500 to the Present Day* (Cambridge and Malden: Polity Press, 2004), 118–42; G. Rosen, *From Medical Police to Social Medicine. Essays on the History of Health Care* (New York: Science History Publications, 1974); Wall, *Bacteria in Britain.*

14 J. A. Mendelsohn, ‘From Eradication to Equilibrium: How Epidemics Became Complex after World War I’, in C. W. Lawrence and G. Weisz, eds, *Greater Than The Parts Holism in Biomedicine 1920-1950* (Oxford and New York: Oxford University Press, 1998), 303–31; J. A. Mendelsohn, ‘Cultures of Bacteriology: Formation and Transformation of a Science in France and Germany, 1870-1914’ (unpublished PhD thesis, Princeton, 1996); P.-O. Méthot, ‘Bacterial Transformation and the Origins of Epidemics in the Interwar Period: The Epidemiological Significance of Fred Griffith’s “Transforming Experiment”’, *Journal of the history of Biology*, 2016, 49, 311–58; C. Kirchhelle, *Typhoid. The Past, Present, and Future of an Ancient Disease* (London: Scala, 2022), 25–31; Judith Walzer Leavitt, ‘“Typhoid Mary” Strikes Back. Bacteriological Theory and Practice in Early Twentieth-Century Public Health’, *Isis*, 1992, 83, 608–29.

15 Wall, *Bacteria in Britain*; Hardy, *Salmonella*, 111–20; Harrison, *Disease and Modern World*, 129–42; P. Chakrabarti, *Bacteriology in British India*, 61–5; R. Rogaski, *Hygienic Modernity. Meanings of Health and Disease in Treaty-Port China* (Berkeley, CA: University of California Press, 2004),136–92; M. Harrison, ‘“The Tender Frame of Man”—Disease, Climate, and Racial Difference in India and the West Indies, 1760-1860’, *Bulletin of the History of Medicine*, 1996, 70, 68–93; M. Echenberg, *Plague**Ports: The Global Urban Impact**of**Bubonic Plague, 1894-1901* (New York: NYU Press, 2010).

16 S. Berger, ‘“Die Jagd auf Mikroben hat erheblich an Reiz verloren”—Der sinkende Stern der Bakteriologie in Medizin und Gesundheitspolitik der Weimarer Politik’, in M. Lengwiler and J. Madarász, eds, *Das**Preventive**Selbst. Eine Kulturgeschichte**Moderner**Gesundheitspolitik* (Bielefeld: Transcript Verlag, 2010), 87–114; Hardy, *Salmonella*, 111–33; C. Kirchhelle, ‘The Forgotten Typers: The Rise and Fall of Weimar Bacteriophage-Typing (1921–1935)’, *Notes and Records*, 2020, 74, 539–565.

17 A. Hardy, ‘Scientific Strategy and Ad Hoc Response: The Problem of Typhoid in America and England, c. 1910–50’, *Journal of the History**of**Medicine**and**Allied Sciences*, 2014, 69, 3–37, 26–29, 32.

18 B. Pouget, ‘*Reflections**on**Medical* *and**Sanitary Action**in**Times**of**Scientific Uncertainty—The Management of the Flu Epidemics by the French Military Medical Service after the World Pandem*ic’, *ESTS* (under review); Mendelsohn, ‘From Eradication to Equilibrium’; Méthot, ‘Bacterial Transformation and the Origins of Epidemics in the Interwar Period’; S. Berger, *Bakterien in Krieg und Frieden: Eine Geschichte der medizinischen Bakteriologie in Deutschland, 1890-1933* (Göttingen: Wallstein Verlag, 2009), 291–374; O. Amsterdamska, ‘Medical and Biological Constraints: Early Research on Variation in Bacteriology’, *Social Studies of Science*, 1987, 17, 657–87; O. Amsterdamska, ‘Stabilizing Instability: The Controversy over Cyclogenic Theories of Bacterial Variation during the Interwar Period’, *Journal of the History of Biology*, 1991, 24, 191–222; C. Gradmann, M. Harrison, and A. Rasmussen, ‘Typhoid and the Military’, *Clinical Infectious Diseases*, 2019, 69, S385–87; R. Davidson, ‘“Searching for Mary, Glasgow”: Contact Tracing for Sexually Transmitted Diseases in Twentieth-Century Scotland’, *Social History of Medicine*, 1996, 9, 195–214.

19 This ideal of networked standardisation was shared at the international level, P. M. H. Mazumdar, ‘The State, the Serum Institutes and the League of Nations’, in C. Gradmann and J. Simon, eds, *Evaluating and Standardizing Therapeutic Agents, 1890-1950* (New York and Basingstoke: Palgrave Macmillan, 2010), 118–38; P. M. H. Mazumdar, ‘In the Silence of the Laboratory’, 437–59.

20 Hardy, *Salmonella Infections*, 114.

21 G. S. Wilson, ‘Public Health Laboratory Service—Part II’, *British Medical Journal*, 1948, 1, 677–82, 678.

22 Hardy, *Salmonella Infections*, 111–30; Wall, *Bacteria in Britain*, 155–76; Hardy, ‘Scientific strategy and Ad Hoc Response’.

23 J. Steere-Williams, *The Filth Disease: Typhoid Fever and the Practices**of**Epidemiology in Victorian England* (Rochester: University of Rochester Press, 2020), 271–82.

24 D. Rowland, *Mapping Communicable Disease Control Administration In The UK. Between Devolution and Europe* (London: Nuffield Trust, 2006), 18.

25 B. Balmer, *Britain and Biological Warfare* (Basingstoke: Palgrave Macmillan, 2001); F. Lentzos, J.-P. Gouyon, and B. Balmer, ‘Imagining Future Biothreats. The Role of Popular Culture’, in A. Wenger *et al*., eds, *The Politics and Science of Prevision* (London and New York: Routledge, 2020), 158–76; M. B. Barry, ‘The Curious Case of the “Sinn Fein Typhoid Plot”’, *History Ireland*, 2020, 6, 30–4.

26 G. S. Wilson, ‘Public Health Laboratory Service—Part II’.

27 R. E. O. Williams, *Microbiology for the Public Health. The Evolution of the Public Health Laboratory Service 1939-1980* (London: Public Health Laboratory Service, 1985), 10 & 19; C. Kirchhelle, Z. A. Dyson, and Dougan, ‘A Biohistorical Perspective of Typhoid and Antimicrobial Resistance’, *Clinical Infectious Diseases*, 2019, 69, S388–94, S390.

28 Hardy, *Salmonella Infections*, 129–33; Williams, *Microbiology for the Public Health*, 60–1; Kirchhelle, Dyson, and Dougan, ‘A Biohistorical Perspective’; M. J. Sikorski and M. M. Levine, ‘Reviving the “Moore Swab”: A Classic Environmental Surveillance Tool Involving Filtration of Flowing Surface Water and Sewage Water to Recover Typhoidal Salmonella’, *Applied and Environmental Microbiology*, 2020, 86, e00060–20.

29 Williams, *Microbiology for the Public Health*, 59–65; K. Hillier, ‘Babies and Bacteria: Phage Typing Bacteriologists, and the Birth of Infection Control’, *Bulletin of the History of Medicine*, 2006, 80, 733–61; F. Condrau, R. G. W. Kirk, ‘Negotiating Hospital Infections: The Debate between Ecological Balance and Eradication Strategies in British Hospitals, 1947-1969’, *Dynamis*, 2011, 31, 385–404; C. Kirchhelle, ‘Swann Song. British Antibiotic Regulation in Livestock Production (1953–2006)’, *Bulletin of the History of Medicine*, 2018, 92, 317–50; M. T. Parker, ‘Clinical Pathology in General Practice: The Public Health Laboratory Service’, *British Medical Journal*, 1953, 2, 823–25; Anonymous, ‘Colindale. Hub of the Public Health Laboratory Service’, *The Lancet*, 1953, 1, 190–91.

30 Rowland, *Mapping Communicable Disease Control Administration in the UK*, 20.

31 Topley and Wilson had studied questions of herd immunity and virulence shifts during the 1920s, for an overview of publications see, M. Greenwood, ‘William Whiteman Carlton Topley, 1886-1944’, *Biographical Memoirs of Fellows of the Royal Society*, 1944, 698–712.

32 G. Pollock, F*evers and Cultures. Lessons for Surveillance, Prevention**and**Control* (Abingdon: Radcliffe Medical Press, 2003), 26; D. Tyrell, ‘William Charles Cockburn’, *Munk’s Roll* (*Royal College of Physicians)*, X, 68.

33 R. O. Valdiserri, ‘Temples of the Future: An Historical Overview of the Laboratory's Role in Public Health Practice', *Annual Review of Public Health*, 1993, 14, 635–648; IOM, *The Future of Public Health. Committee for the Future of Public Health* (Washington DC: Institute of Medicine, 1988), 67.

34 Valdiserri, ‘Temples of the Future', 641; CDC, ‘Achievements in Public Health, 1900-1999: Changes in the Public Health System', *MMWR*, 1998, 48, 1141–1147; Anonymous, ‘Public Health Laboratories', *JAMA*, 1916, 66, 1309–1310. S. B. Thacker and R. L. Berkelman, ‘Public Health Surveillance in the United States’, *Epidemiologic Reviews*, 1988, 10, 164–90, 165; J. Farley, *To Cast Out Disease: A History of the International Health Division of Rockefeller Foundation (1913-1951)* (Oxford: Oxford University Press, 2004).

35 D. Satcher, ‘The History of the Public Health Service and the Surgeon General's Priorities’, *Food and Drug Law Journal*, 1999, 54, 13–19, 14–15.

36 Satcher, ‘The History of the Public Health Service’, 15; ‘Notifiable Disease Surveillance and Notifiable Disease Statistics—United States, June 1946 and June 1996’, *MMWR*, 1996, 45, 531.

37 Valdiserri, ‘Temples of the Future’, 645–46; L. Wyatt, *Intergovernmental Relations in Public Health* (Minnesota: University of Minnesota Press, 1951), 6–11.

38 J. R. Wright Jr, ‘The Politics Underlying the Provision of and Changes in Pathology and Laboratory Services in the United States during the Roaring Twenties’, *Archives of Pathology & Laboratory Medicine*, 2016, 140, 983–91.

39 J. P. Leider *et al.*, ‘How much do we spend? Creating historical estimates of public health expenditures in the United States at the federal, state, and local levels’, *Annual Review of Public Health*, 2018, 39, 471–87, 473–474.

40 J. W. Mountin and E. E. Flook, *Distribution of Health Services in the Structure**of**State Government* (Washington DC: US Government Printing Office, 1947), 16 [page numbers based on total pages in volume].

41
*Ibid*., 43 and 57.

42
*Ibid*., 313–16.

43
*Ibid*., 18.

44 T. F. Whayne, ‘The History of Preventive Medicine in World War II’, *Public Health Reports*, 74, 1959, 170; R. S. Anderson *et al.*, eds, *Preventive Medicine in World War* II, Vol. 9 (Washington DC: Office of the Surgeon General Department of the Army, 1969), 256–78; ‘Essay—Public Health and War’, *US National Library of Medicine—History of Medicine*, 12 October 2021. https://www.nlm.nih.gov/hmd/digicolls/phfgtw/essay.html (accessed 26 April 2022); A. K. McVety, *The**Rinderpest**Campaigns:**A**Virus, Its Vaccines*, *and**Global Development in the Twentieth Century* (Cambridge: Cambridge University Press, 2018), 47–85.

45 CDC, ‘Achievements in Public Health, 1900-1999’, 1145.

46 Pollock, *Fevers and Cultures*, 35.

47
*Ibid*., 36; S. B. Thacker, J. R. Qualters, L. M. Lee, ‘Public Health Surveillance in the US: Evolution and Challenges’, *MMWR*, 2012, 61, 3–9; less comprehensive predecessor reports had been published by the PHS since 1930 (*Weekly Health Index* (1930–41), Weekly Mortality Index (1941–52), Morbidity and Mortality (1952–75), Morbidity and Mortality Weekly Report (1976–present).

48 E. W. Etheridge, *Sentinel for Health: A History of the Centers for Disease Control* (Berkeley: University of California Press, 1992), 36–86; E. Gosselin, *Constructing International Health: The Communicable Disease Center, Field Epidemiologists and the Politics of Foreign Assistance (1948-1972)* (Montreal: McGill University, PhD, 2011), 56–7; C. Kirchhelle, ‘Between Bacteriology and Toxicology—Agricultural Antibiotics and US Risk Regulation’, in A. Creager, J-P. Gaudillière, eds, *Risk on the Table* (New York: Berghahn, 2021), 214–242, 217–18.

49 McVety, *Rinderpest**Campaigns*, 175–190; E. Conis, *Vaccine Nation. America’s Changing Relationship**with**Immunization* (Chicago and London: University of Chicago Press, 2015), 4–9.

50 E. J. Tiffany, ‘Public Health and Clinical Laboratories in the Diagnosis of Enteric Bacterial Infections’, *Public Health Reports*, 1952, 67, 1069–75; CDC, *History of The National Communicable Disease Centre From 1963 to 1968* (Atlanta: Department of Health, Education, and Welfare—Public Health Service, 1969); CDC, ‘History of CDC’, *MMWR*, 1996, 45, 526.

51 CDC, History *of the National Communicable Disease Center*, 33.

52 H. M. Marks, ‘Review of Sentinel for Health’, *Journal of Southern History*, 60, 1994, 170.

53 Quoted according to Etheridge, *Sentinel for Health*, 58.

54 Gosselin, *Constructing International Health*, 56.

55 L. Weir and E. Mykhalovskiy, ‘The Geopolitics of Global Public Health Surveillance in the Twenty-First Century’, in A. Bashford, ed., *Medicine at the Border. Disease, Globalization and Security, 1850 to the Present* (London: Palgrave Macmillan, 2007), 243.

56 A. D. Langmuir, ‘The Surveillance of Communicable Diseases of National Importance’, *New England Journal of Medicine*, 1963, 268, 182–92, 182–183.

57 E. Fee and T. M. Brown, ‘Preemptive Biopreparedness: Can We Learn Anything from History?’ *American Journal of Public Health*, 91, 2001, 721–26; Gosselin, *Constructing International Health*, 62–68; L. Fearnley, ‘Epidemic Intelligence. Langmuir and the Birth of Disease Surveillance’, *BEHEMOTH-A Journal on Civilisation*, 3, 2010, 36–56.

58 Etheridge, *Sentinels*, 58.

59 Gosselin, *Constructing International Health*, 171–229.

60 F. Allerberger, ‘Strukturelle Voraussetzungen und Bedingungen für eine effektive mikrobiologische Diagnostik bei Ausbruchsgeschehen’, *Bundesgesundheitsblatt-Gesundheitsforschung-Gesundheitsschutz*, 2013, 56, 22–7, 22.

61 M. Thießen, *Die Immunisierte Gesellschaft.* (Göttingen: Vandehoeck & Ruprecht, 2017), 138; J. Vossen, *Gesundheitsämter im Nationalsozialismus* (Düsseldorf: Klartext Verlag, 2001), 218–19.

62 J. Vossen, ‘Tuberkulosefürsorge in Deutschland 1900 bis 1945’, in M. Forßbohm *et al*., eds, *Handbuch Tuberkulose für Fachkräfte an Gesundheitsämtern* (Düsseldorf: Akademie für öffentliches Gesundheitswesen in Düsseldorf, 2009), 33.

63 A. Labisch and W. Woelk, ‘Öffentliche Gesundheit in der Nachkriegsgeschichte der Bundesrepublik Deutschland', *Public Health Forum* (1996) 4, 3–4; Berger, ‘Die Jagd auf Mikroben hat erheblich an Reiz verloren', 105–6; A. Hüntelmann, ‘Biopolitische Netzwerke: Die interpersonellen und interinstitutionellen Verbindungen zwischen dem Institut für Infektionskrankheiten und dem Reichsgesundheitsamt vor 1935’, in M. Hulverscheidt and A. Laukötter, eds, *Infektion und Institution Zur Wissenschaftsgeschichte des Robert Koch-Instituts im Nationalsozialismus* (Göttingen: Wallstein Verlag, 2009), 42–66; A. Hinz-Wessels, ‘Das RKI unter der NS-Diktatur. Personelle, administrative und inhaltliche Umgestaltung zwischen 1933 und 1945’, in M. Hulverscheidt and A. Laukötter, eds, *Infektion und Institution Zur Wissenschaftsgeschigte des Robert Koch-Instituts im Nationalsozialismus* (Göttingen: Wallstein Verlag, 2009), 67–88.

64 R. Neuhaus and W. F. Schräder, ‘Planning and Management of Public Health in the Federal Republic of Germany’, *Health Policy*, 1985, 5, 99–109, 100; U. Lindner, *Gesundheitspolitik in der Nachkriegszeit: Grossbritannien und die Bundesrepublik Deutschland im Vergleich* (München: Oldenbourg Verlag, 2004), 62-5, 70.

65 From 1969 to 1986 Federal Ministry of Youth, Family and Health, from 1986 to 1991 Federal Ministry for Youth, Family, Women, and Health. Responsibility for the statutory health insurance system was shared with the Federal Ministry of Labour and Social Affairs.

66 R. Neuhaus and W. F. Schräder, ‘Planning and Management of Public Health in the Federal Republic of Germany’, 100–101; K. Plümer, ‘Germany’, in B. Rechel *et al*., eds, *Organization and Financing**of**Public Health Services in Europe: Country**Reports* (Copenhagen: WHO Regional Office for Europe, 2018), 35–48; Lindner, *Gesundheitspolitik in der Nachkriegszeit*, 13–31.

67 Lindner, *Gesundheitspolitik in der Nachkriegszeit*, 62–5.

68 Neuhaus and Schräder, ‘Planning and Management of Public Health in the Federal Republic of Germany’, 105; Lindner, *Gesundheitspolitik in der Nachkriegszeit*, 70-1, 109.

69 M. Thießen, ‘Vergleichende, verfeindete und verflochtene Gesellschaften. Transnationale Zusammenhänge einer bundesdeutschen Geschichte der Gesundheit’, in M. Kohlrausch *et al.*, eds, *Wo liegt die Bundesrepublik? Vergleichende Perspektiven auf die westdeutsche Geschichte* (Göttingen: Vandenhoeck & Ruprecht, 2016), 130–37; M. Thießen, ‘Vorsorge als Ordnung des Sozialen: Impfen in der Bundesrepublik und der DDR’, *Zeithistorische Forschungen*, 2013, 10, 409–32; *Health Care Systems in Transition*: *Germany* (European Observatory on Health Care Systems, 2000), 15; J.D.G. Poldermans, ‘Statistics and planning in the health services of West Germany and the United Kingdom', *Social Science & Medicine*, 1971, 5, 339-61

70 Neuhaus and Schräder, ‘Planning and Management of Public Health in the Federal Republic of Germany’, 101.

71 Lindner, *Gesundheitspolitik in der Nachkriegszeit*, 70–1.

72 Neuhaus and Schräder, ‘Planning and Management of Public Health in the Federal Republic of Germany’, 106.

73 This insularity also impacted the development of West German vaccine schedules; Thießen, *Immunisierte Gesellschaft*, 260–61.

74 K. Plümer, ‘Germany’, 36–40.

75 Neuhaus and Schräder, ‘Planning and Management of Public Health in the Federal Republic of Germany’, 102; Lindner, *Gesundheitspolitik in der Nachkriegszeit*, 30.

76 Etheridge, *Sentinel for Health*, 101–03; R. Packard, *A History of Global Health: Interventions into the Lives of Other Peoples* (Baltimore: Johns Hopkins Press, 2016), 105–31; C. Kirchhelle and C. Kirchhelle, ‘Northern Normal. Laboratory Networks, Microbial Culture Collections, and Taxonomies of Power (1939-2000)’, *ESTS* (under review).

77 M. Lengwiler, J. Madarász, ‘Präventionsgeschichte als Kulturgeschichte der Gesundheitspolitik’, in M. Lengwiler and J. Madarász, eds, *Das präventive Selbst Eine Kulturgeschichte moderner Gesundheitspolitik* (Bielefeld: Transcript Verlag, 2010), 11–28; C. Timmermann, ‘Risikofaktoren: Der scheinbar unaufhaltsame Erfolg eines Ansatzes aus der amerikanischen Epidemiologie in der deutschen Nachkriegsmedizin’, in M. Lengwiler and J. Madarász, eds, *Das präventive Selbst Eine Kulturgeschichte moderner Gesundheitspolitik* (Bielefeld: Transcript Verlag, 2010), 251–77.

78 Thacker and Berkelman, ‘Public Health Surveillance in the US’, 173.

79 J. Howie, ‘The Public Health Laboratory Service’, *The Lancet*, 1965, 285, 501–05.

80
*Public Health in England. The* *Report* *of the Committee of Inquiry into the Future Development of the Public Health Function* (London: House of Commons, 1988), 5–6.

81 M. Gorsky, K. Lock, and S. Hogarth, ‘Public Health and English Local Government: Historical Perspectives on the Impact of “Returning Home”’, *Journal of Public Health*, 2014, 36, 546–51, 548; Lindner, *Gesundheitspolitik in der Nachkriegszeit*, 81.

82 Lindner, *Gesundheitspolitik in der Nachkriegszeit*, 81.

83 Gorsky, Lock, and Hogart, ‘Public Health and English Local Government’; R. Baggott, *Public Health. Policy and Politics*, 2nd edn (Houndsmill & New York: Palgrave Macmillan, 2000), 46–7.

84 Pollock, *Fevers and Cultures*, 27.

85 Williams, *Microbiology for the Public Health*, 86.

86 W. M. Edgar, ‘Letters to the Editor: The Public Health Laboratory Service’, *The Lancet*, 1965, 285, 599; A. J. N. Warrack, ‘Clinical Pathology in General Practice’, *BMJ*, 1953, 2, 935; M. T. Parker, ‘Clinical Pathology in General Practice’, *BMJ*, 1953, 2, 1048.

87 Williams, *Microbiology for Public Health*, 84–5.

88
*Ibid*., 94.

89 P. Clark, ‘“Problems of Today and Tomorrow”: Prevention and the NHS in the 1970s’, *Social History of Medicine*, 2020, 33, 981–1000.

90 Williams, *Microbiology for Public Health*, 142–43.

91 Pollock, *Fevers and Cultures*, 27.

92 N. S. Galbraith, ‘CDSC: From Cox to Acheson’, *Journal of Public Health*, 1989, 11, 187–99.

93 Williams, *Microbiology for the Public Health*, 119; Nuffield Trust, 1968-1977: Rethinking the NHS, *The History of the NHS*, https://www.nuffieldtrust.org.uk/chapter/1968-1977-rethinking-the-national-health-service-1 (accessed 26 April 2022).

94 Williams, *Microbiology for the Public Health*, 135-6; N. S. Galbraith, ‘CDSC: From Cox to Acheson’, 188.

95
*Ibid*., 190.

96
*Ibid*., 188.

97 Williams, *Microbiology for the Public Health*, 119; Pollock, *Fevers and Cultures*, 28–9; ‘Nuffield Trust, 1968-1977: Rethinking the NHS’, *The History of the NHS*, 15 October 2021, https://www.nuffieldtrust.org.uk/chapter/1968-1977-rethinking-the-national-health-service-1 (accessed 26 April 2022).

98
*Public Health in England*, 17.

99 Williams, *Microbiology for the Public Health*, 120, 125, and 174.

100
*Report of the DHSS review of the Public Health Laboratory Service* (London: Department of Health and Social Security, 1985); J. T. Macfarlane and M. Worboys, ‘Showers, Sweating and Suing: Legionnaires’ Disease and ‘New’ Infections in Britain, 1977–90’, *Medical History*, 2012, 56, 72–93, 76 & 82.

101 J. Howie, ‘Threat to the PHLS’, *BMJ*, 1985, 290, 579-80.

102
*Ibid*.; G. Turner, ‘Threat to the PHLS’, *BMJ*, 1985, 290, 787; R. A. Hughes, ‘PHLS reform. Professional advisers' views over-ruled’, *BMJ*, 1985, 291, 616; A. Veitch, ‘Shake-up “will harm disease detection”’, *Guardian*, 08 March 1985; *Lords Sitting of Wednesday, 20th November, 1985* (London: Hansard, 1985); *House of Lords—Public Health Laboratory Service: 04**June**1985* (London: Hansard, 1985).

103 ‘PHLS celebrates Golden Jubilee', *Lancet* 1996, 348, 1371; D. Miller, ‘Risk, Science and Policy: Definitional Struggles, Information Management, The Media and BSE’, *Social Science & Medicine*, 1999, 49, 1239–55.

104 Etheridge, *Sentinel*, 188–223; D. Schneider *et al.*, ‘Training the Global Public Health Workforce through Applied Epidemiology Training Programs: CDC’s experience, 1951–2011’, *Public Health Reviews*, 2011, 33, 190–203; S. Bhattacharya, ‘Reflections on the Eradication of Smallpox’, *The Lancet*, 2010, 375, 1602–03; Gosselin, *Constructing International Health*, 107–08.

105 Gosselin, *Constructing International Health*, 43 & 68.

106 R. M. Schmidt and M. A. Madoff, ‘The State and Territorial Public Health Laboratory: Program Activities, Organization and Prospects for the Future’, *American Journal of Public Health*, 1977, 67, 433–38, 433.

107
*Consolidated Annual Report on State and Territorial Public Health Laboratories Fiscal Year 1982* (Atlanta: HHS Publication, 1984), 5.

108
*Ibid*., 6.

109
*Ibid*.; *Consolidated Annual Report On State & Territorial Public Health Laboratories Fiscal year 1975* (Atlanta: CDC, 1976), 6.

110
*Consolidated Annual Report on State and Territorial Public Health Laboratories Fiscal Year 1982*, 3.

111
*Public Health Personnel in the United States, 1980* (Washington: HHS, 1982), 234–5.

112 Schmidt and Madoff, ‘The State and Territorial Public Health Laboratory’, 436–7; D. Adcock and H. Lawton, ‘The Paperwork Crunch in State Public Health Laboratories’, *AJPH*, 1981, 71, 748–50.

113 Etheridge, *Sentinel*, 224–34.

114 R. L. Berkelman and P. Freeman, ‘Emerging Infections and the CDC Response’, in R. Packard *et al*., eds, *Emerging Illnesses and Society: Negotiating the Public Health Agenda* (Baltimore: Johns Hopkins University Press, 2004), 350–87; on the wider contemporary orientation towards risk factors see: C. Timmermann, ‘Risikofaktoren: Der scheinbar unaufhaltsame Erfolg eines Ansatzes aus der amerikanischen Epidemiologie in der deutschen Nachkriegsmedizin’.

115 Etheridge, *Sentinel*, 247–76; Berkelman and Freeman, ‘Emerging Infections and the CDC Response’.

116 L. Etheredge, ‘Reagan, Congress, and health spending’, *Health Affairs*, 1983, 2, 14–24.

117 Etheridge, *Sentinel for Health*, 310–40; ‘Microscope on Washington’, *Laboratory Medicine*, 1982, 13, 76–77; between fiscal year of 1981 and 1988, 62 staff-years were reduced for lab activities and 82 staff years for other areas of infectious disease control; *CDC Control Staffing for AIDS and Other Programs* (Washington DC: GAO, 1989), 4.

118 Ibid.

119 E. Smith, ‘An Overview of Venereal Disease Programs in Britain, West Germany, Denmark, and Sweden, With Implications for Canada’, *Bulletin of the Pan American Health Organization (PAHO)*, 1976, 10, 321–32.

120 K. Meyer-Hartwig and W. Bleifeld, ‘Education and Professional Training of Biomedical Engineers in West Germany’, *Journal of Medical Engineering & Technology*, 1978, 2, 62–6.

121 Neuhaus and Schräder, ‘Planning and Management’, 102–03; Plümer, ‘Germany’, 36*; Health Care Systems in Transition*: *Germany*, 15–18.

122
*Ibid*., 57–58; Plümer, ‘Germany’, 36.

123
*Health Care Systems in Transition*: *Germany*, 58.

124 Neuhaus and Schräder, ‘Planning and Management’, 101.

125 U. Frank *et al.*, ‘The Organization of Infection Control in Germany’, *Journal of Hospital Infection*, 2001, 49, 9–13, 9–10.

126 J.W. Hurst. ‘Reform of Health Care in Germany’, *Health Care Financing Review* 1991, 12(3), 73–80, 73 and 78.

127 R. Reintjes *et al.*, ‘Infectious Diseases Before and After German Unification: Trends in Mortality and Morbidity’, *European Journal of Epidemiology*, 2001, 17, 1105–10, 1105–6.

128 See, for example: J. T. Crane, *Scrambling for Africa: AIDS*, *Expertise, and the**Rise**of American Global Health Science* (Ithaca: Cornell University Press, 2013); Packard, *Global Health*; I. N. Okeke, *Divining Without Seeds: The Case For Strengthening Laboratory Medicine in Africa* (Ithaca: Cornell University Press, 2011); Lakoff, *Unprepared*; Koster, ‘An Oral History of medical laboratory development in francophone West African countries'.

129 V. Berridge, D. Christie, and E. Tansey, *Public Health in the 1980s and 1990s: Decline and Rise?* (London: Wellcome Trust Centre for the History of Medicine at UCL, 2006), xxi–xxv.

130
*Public Health in England*, 7–8, 66–71; Baggott, *Public Health*, 46–9.

131
*Public Health in England*, 46.

132
*Ibid*., 17.

133
*Ibid*., 45; S. Kisely and J. Jones, ‘Acheson Revisited: Public Health Medicine Ten Years after the Acheson Report’, *Public Health*, 1997, 111, 361–64.

134
*Public Health in England*, 46.

135
*Ibid*., 53-4.

136 Pollock, *Fevers and Cultures*, 30.

137 Kisely and Jones, ‘Acheson revisted’.

138 B. Duerden, ‘Twenty-First-Century Medical Microbiology Services in the UK’, *Nature Reviews Microbiology*, 2005, 3, 979–83; U. Lindner, ‘Die Krise des Wohlfahrtsstaats im Gesundheitssektor Bundesrepublik Deutschland, Großbritannien und Schweden’, *Archiv für Sozialgeschichte*, 2007, 47, 297–324, 316–8; *PHLS: Statement of Achievements* (London: PHLS, 1995).

139 C. J. Lahti, ‘Pulsed Field Gel Electrophoresis in the Clinical Microbiology Laboratory’, *Journal of Clinical Laboratory Analysis*, 1996, 10, 326–30; *A Report of the Study of Infectious Intestinal Disease in England* (London: Food Standards Agency/Public Health Laboratory Service, 2000), 18–19; C. Verity, D. Manning, and A. Nicoll, ‘Consent, Confidentiality, and the Threat to Public Health Surveillance’, *BMJ*, 2002, 324, 1210–13.

140 Pollock, *Fevers and Cultures*, 31.

141
*Ibid*., 32; S. Borriello, ‘Near Patient Microbiological Tests’, *BMJ*, 1999, 319, 298–301; C. Kirchhelle, *Pyrrhic Progress: Antibiotics in Anglo-American Food Production (1949-2018)* (New Brunswick: Rutgers University Press, 2020), 274–6.

142 Rowland, *Mapping Communicable Disease Control*, 20.

143 P. Pini, ‘PHLS Celebrates Golden Jubilee’, *The Lancet*, 1996, 348, 1371.

144 Berkelman and Freeman, ‘Emerging Infections and the CDC Response', 353-8.

145 IoM, *The Future of Public Health,* 183.

146
*Ibid*., 181.

147
*Ibid*., 188.

148 Berkelman and Freeman, ‘Emerging Infections and the CDC Response’, 353.

149 IoM, *The Future of Public Health*, 19.

150
*Ibid*., 176.

151
*Ibid*., 114.

152
*Ibid*., 8.

153
*Ibid*., 15.

154 IoM, *Emerging Infections. Microbial Threats to Health in the United States* (Washington DC: Institute of Medicine, 1992), 3.

155
*Ibid*., 12.

156
*Ibid*., 6.

157
*Ibid*., 4.

158 N. B. King, ‘The Scale Politics of Emerging Diseases’, *Osiris*, 2004, 19, 62–76; A. Lakoff, ‘The generic Biothreat, or, How We Became Unprepared’, *Cultural Anthropology*, 2008, 23, 412–13; M. Harrison, ‘Pandemics’, in M. Jackson, ed., *The Routledge History of Disease* (Abingdon: Routledge, 2017), 137; Scott H. Podolsky, ‘The Evolving Response to Antibiotic Resistance (1945–2018)’, *Palgrave Communications*, 2018, 4, 1–8; Lakoff, *Unprepared*, 78.

159 Christopher Long, *The Molecularisation of Security: Medical Countermeasures, Stockpiling and the Governance of Biological Threats* (London: Routledge, 2021).

160 F. Brauer, ‘Mathematical Epidemiology: Past, Present, and Future’, *Infectious Disease Modelling*, 2, 2017, 113–27; Engelmann, ‘A box, a trough and marbles’, 1-24; Harrison, ‘Pandemics', 133.

161 A. Lakoff, ‘The Generic Biothreat’, 399–428; C. Caduff, *The Pandemic Perhaps*; A. Lakoff, *Unprepared*, 45-66.

162 Thacker and Berkelman, ‘Public Health Surveillance in the US’, 182; S. Podolsky, *The Antibiotic Era. Reform, Resistance, and the Pursuite of a Rational Therapeutics* (Baltimore: Johns Hopkins University Press, 2015), 172.

163 Pollock, *Fevers and Cultures*, 38.

164 IOM, *Emerging Infections*, 119-20; ‘Public Health Laboratory Information System', *CDC Wonder - Scientific Data Documentation*; https://wonder.cdc.gov/wonder/sci_data/misc/type_txt/phlis.asp (accessed: 28.04.2022); see, for example, the slow adoption of electronic reporting in the case of AMR; F. Perez and M. V. Villegas, ‘The Role of Surveillance Systems in Confronting the Global Crisis of Antibiotic-Resistant Bacteria’, *Current**Opinion**in Infectious Diseases*, 2015, 28, 375-83

165 Pollock, *Fevers and Cultures*, 17.

166 R. L. Berkelman *et al.*, ‘Infectious Disease Surveillance: A Crumbling Foundation’, *Science*, 1994, 264, 368–70.

167
*Ibid*., 368.

168 W. R. Dowdle, ‘The Future of the Public Health Laboratory’, *Annual Review of Public Health*, 1993, 14, 649–64, 656.

169 A. Lakow, *Unprepared. Global Health in a Time of Emergency* (Berkeley: Univerity of California Press, 2017), 142–44.

170 Berkelman et al., ‘Infectious disease surveillance’, 368-9;

171 R. U. Fishman, ‘What is APHA's Future in Public Health?’, *Journal of Public Health Policy*, 1992, 13, 14–17, 15.

172 CDC, ‘Estimated Expenditures for Core Public Health Functions—Selected States, October 1992–September 1993’, *MMWR*, 1995, 44, 421, 27.

173 CDC, ‘Estimated Expenditures for Essential Public Health Services—Selected States, Fiscal Year 1995’, *MMWR*, 1997, 46, 150.

174
*Report of the Review of NHS Pathology Services in England. Chaired by Lord Carter of Coles. An Independent Review for the Department of Health* (London, 2006), 65.

175 IoM, *The Future of Public Health*, 89; Dowdle, ‘The Future of the Public Health Laboratory’, 655.

176 Y. M. Felman, M. C. Corsaro, and J. R. Jones, ‘Laboratory Compliance with Syphilis Reporting Laws: The New York City Experience 1972-77’, *Public Health Reports*, 1980, 95, 53.

177 G. Avery, ‘Outsourcing Public Health Laboratory Services: A Blueprint for Determining Whether to Privatize and How’, *Public Administration Review*, 2000, 60, 330–37.

178 Dowdle, ‘The Future of the Public Health Laboratory’, 656.

179 Avery, ‘Outsourcing Public Health Laboratory Services’; K. M. Gebbie, ‘Public Health’, in R. R. Nelson, ed., *The Limits of Market Organisation* (New York: Russell Sage Foundation, 2005), 321–34.

180 Dowdle, ‘The Future of the Public Health Laboratory’, 653–8.

181 Ibid., 660; Avery, ‘Outsourcing Public Health Laboratory Services’, 334, 336.

182 Berkelman *et al.*, ‘Infectious Disease Surveillance', 368; W. L. Roper *et al.*, ‘Strengthening the Public Health System’, *Public Health Reports*, 1992, 107, 609-15; ‘Laboratory Capacity to Detect Antimicrobial Resistance, 1998’, *MMWR*, 2000, 48, 1167; J. I. Tokars *et al.*, ‘US Hospital Mycobacteriology Laboratories: Status and Comparison with State Public Health Department Laboratories’, *Journal of Clinical Microbiology*, 1996, 34, 680–85; B. Swaminathan *et al.*, ‘PulseNet: the Molecular Subtyping Network for Foodborne Bacterial Disease Surveillance, United States’, *Emerging Infectious Diseases*, 2001, 7, 382-9.

183 B. Steinhardt, *Emerging Infectious Diseases. Consensus on Needed Laboratory Capacity Could Strengthen Surveillance* (Washington DC: General Accounting Office, 1999), 21–32.

184 D. B. Epstein, ‘Recommendations for a Regional Strategy for the Prevention and Control of Emerging Infectious Diseases in the Americas’, *Emerging Infectious Diseases*, 1995, 1, 103.

185 L.D. Brown and V.E. Amelung, ‘‘Manacled Competition’: Market Reforms In German Health Care’, *Health Affairs*, 1999, 18(3), 76-91.

186 Plümer, ‘Germany’, 36; K. Riedmann, ‘Die historische Entwicklung der Gesundheitsberichterstattung in Deutschland’, *Bundesgesundheitsblatt-Gesundheitsforschung-Gesundheitsschutz*, 2000, 43, 594–99.

187 Plümer, ‘Germany’, 36.

188 R. Busse and M. Blümel, *Germany: Health System Review* [Health Systems in Transition, 16/2] (Copenhagen: WHO, 2014), 178.

189 Plümer, ‘Germany', 36; Frank *et al.*, ‘The Organization of Infection Control in Germany’, 9.

190 P. D. Weinberg *et al.*, ‘Legal, Financial, and Public Health Consequences of HIV Contamination of Blood and Blood Products in the 1980s and 1990s’, *Annals of Internal Medicine*, 2002, 136, 312–19; *Zweite Beschlußempfehlung und Schlußbereicht des 3. Untersuchungsausschusses nach Artikel 44 des Grundgesetzes*. *Deutscher Bundestag 12. Wahlperiode*, Bonn, 1994.

191
*Ibid*.; S. Dressler, ‘Blood “Scandal” and AIDS in Germany’, in R. Bayer and E. Feldman, eds, *Blood**Feuds: AIDS, Blood, and the Politics Of Medical Disaster* (New York and Oxford: Oxford University Press, 1999), 191–212.

192 G. Laude, M. Kist, and G. Krause, ‘Etablierung von Referenznetzwerken aus Nationalen Referenzzentren mit assoziierten Konsiliarlaboratorien in Deutschland’, *Bundesgesundheitsblatt-Gesundheitsforschung-Gesundheitsschutz*, 2009, 52, 919–26, 919.

193 Frank *et al.*, ‘The Organization of Infection Control in Germany’, 10–13; S. Beermann *et al.*, ‘Public Health Microbiology in Germany: 20 Years of National Reference Centers And Consultant Laboratories’, *International Journal of Medical Microbiology*, 2015, 305, 595–600; L. R. Petersen *et al.*, ‘Developing National Epidemiologic Capacity To Meet the Challenges of Emerging Infections in Germany’, *Emerging Infectious Diseases*, 2000, 6, 576–84; *Health Care Systems in Transition*, 18–19.

194 K. Riedmann, ‘Die historische Entwicklung der Gesundheitsberichterstattung in Deutschland’, 594; R. Busse and M. Blümel, ‘Germany: Health System Review’, 69.

195
*Seuchenrecht Soll Neu Geordnet Werden (Gesetzentwurf)* (Berlin: Deutscher Bundestag, 2000).

196
*Health Care Systems in Transition* (2000), 58.

197 Plümer, ‘Germany’, 40.

198 Rowland, *Mapping Communicable Disease Control*, 27–33.

199 L. Donaldson, *Getting Ahead of the Curve. A Strategy**for**Combating Infectious Diseases (Including Other Aspects**of**Health Protection)* (London, 2002).

200
*Ibid*., 9–14.

201 Rowland, *Mapping Communicable Disease Control*, 27–32; L. Donaldson, *Getting Ahead of the Curve*, 139–140.

202 The Centre for Applied Microbiology and Research (CAMR) at Porton Down had already been part of the PHLS between 1979 and 1994.

203
*The History of the Health Protection Agency 2003-2013* (London: Public Health England, 2013).

204 Rowland, *Mapping Communicable Disease Control*, 28.

205
*Health Protection Agency. Annual Report & Accounts* (London: Health Protection Agency, 2007), 33.

206
*The History of the Health Protection Agency 2003-2013*, 28.

207
*Memorandum by the Public Health Laboratory Service (PHLS). House of Lords Select Committee on Science and Technology* (London: HMSO, 2003), 21-22.

208 See also *The History of the Health Protection Agency 2003-2013*, 31, 36, 51.

209 D. R. Harper, ‘Preparedness for SARS in the UK in 2003’, *Philosophical Transactions of the Royal Society of London Series B: Biological Sciences*, 2004, 359, 1131–32.

210 P. P. Mortimer, ‘Stray Thoughts on SARS’, *Communicable Disease and Public Health*, 2003, 6, 172–76, 173.

211
*The History of the Health Protection Agency 2003-2013*, 26.

212 Ibid., 16; in Wales, former PHLS services were integrated with local public health functions provided by Consultants for Communicable Disease Control. The new locally rooted service became the Communicable Disease Division of the National Public Health Service for Wales and worked closely with NHS microbiology labs, Duerden, ‘Twenty-First-Century Medical Microbiology Services in the UK’, 1.

213
*The History of the Health Protection Agency 2003-2013*, 39-40.

214 Duerden, ‘Twenty-First-Century Medical Microbiology Services in the UK’, 2–4.

215
*Report of the Review of NHS Pathology Services in England. Chaired by Lord Carter of Coles* (London: Department of Health, 2006), 43.

216 Rowland, *Mapping Communicable Disease Control*, 33.

217
*The History of the Health Protection Agency 2003-2013*, 32–3.

218 Ibid., 46 and 55; R. England, ‘The Dangers of Disease Specific Programmes for Developing Countries,’ *BMJ*, 2007, 335, 565–65; C. Cashin et al., *Earmarking for Health. From Theory to Practice* (Geneva: WHO, 2017).

219
*The History of the Health Protection Agency 2003-2013*, 17-18, 46, 55-7, 78-9.

220
*Ibid*., 64–5; 83-5; 92-4.

221 P. Das, ‘Duncan Selbie: The New Face of Public Health in England’, *The Lancet*, 2013, 381, 1175; *Public Health England. Evaluation and Recommendations* (Atlanta: IANPHI, 2017), 8.

222 Ibid., 6.

223 Gorsky, Lock, and Hogarth, ‘Public Health and English Local Government’, 546.

224
*Ibid*., 546, 549–50; J. Middleton and G. Williams, ‘England’, in B. Rechel *et al.*, eds., *Organization and**Financing**of Public Health Services**in**Europe Country Reports* (Copenhagen: WHO Europe, 2018), 5–22.

225
*Public Health England (PHE). Evaluation and Recommendations*, 5–10; *Summary of updated health protection legislation (England)* (London: Chartered Institute of Environmental Health, 2010).

226
*The History of the Health Protection Agency 2003-2013*, 108.

227
*Health and Local Public Health Cuts, House of Commons Briefing 14 May 2019* (London: Local Government Association, 2019); other estimates speak of an even more dramatic reduction of £700–850 million in net expenditure between 2014/2015 and 2019/2020 with the poorest areas in England experiencing disproportionately high cuts of almost 15 per cent; C. Thomas, *Hitting the Poorest Worst? How Public Health Cuts Have Been Experienced in England’s Most Deprived Communities* (London: Institute for Public Policy Research (IPRR), 2019).

228 J. Middleton and G. Williams, ‘England’, 13–4; L. Marks *et al.*, ‘The *Return**of**Public Health**to**Local Government**in**England: Changing**the**Parameters of the Public Health Prioritization Deb*ate?’ *Public Health*, 2015, 129, 1194–203; *Public Health England (PHE). Evaluation and Recommendations*, 10.

229 J. Middleton and G. Williams, ‘England’, 16.

230 Pollock, *Fevers and Cultures*, 36; B. Steinhardt, *Emerging Infectious Diseases*, 1–10.

231
*Ibid*., 15.

232
*Ibid*., 17.

233
*Ibid*., 18.

234 CDC, *Public Health’s Infrastructure. Every Health Department Fully Prepared; Every Community Better Protected. A Status Report* (Atlanta: CDC, 1999), ii.

235
*Ibid*., iii–v.

236 A. S. Khan, A. M. Levitt, M. J. Sage, ‘Biological and Chemical Terrorism; Strategic Plan For Preparedness and Response: Recommendations of the CDC Strategic Planning Workgroup’*, MMWR*, 2000, 49, 1–14.

237 J. Witt-Kushner *et al.*, ‘Core Functions and Capabilities of State Public Health Laboratories; A Report of the Association of Public Health Laboratories’, *MMWR*, 2002, 51, 1-8; R. M. Davis, ‘Healthy People 2010: Objectives for the United States: Impressive, But Unwieldy’, *BMJ*, 2000, 320, 818–819.

238 IoM, *The Future of The Public’s Health in the 21st Century* (Washington DC: Institute of Medicine, 2003), 3.

239 D. Rosner and G. Markowitz, *Are we Ready? Public Health since 9/11* (Berkeley, CA : University of California Press, 2006), 68; for pre-existing preparedness spending see, *Ibid*., 131–5.

240 J. Witt-Kushner *et al.*, ‘Core Functions and Capabilities of State Public Health Laboratories’; B. J. Turnock, *Public Health Preparedness At A Price*: *Illinois* (New York: Century Foundation, 2004), 10–13; K. C. Milne and T. L. Milne, ‘Public Health Laboratory System Improvement Program: Development and Implementation’, *Public Health Reports*, 2010, 125, 31–39; Rosner and Markowitz, *Are we Ready?*, 106–16.

241
*Ibid*., 57–75; CDC, *Public Health Preparedness: Mobilizing State by State* (Atlanta: CDC, 2008), 1-5; 12; 15-21; *APHL Report, Data Summary of the 2010 Core Laboratory Profiles Survey* (APHL, 2011), 5; M.L. Wilson et al., ‘The Role of Local Public Health Laboratories', *Public Health Reports*, 2010, 125 (Spl 2), 118–122, 119.

242 D. Boxrud *et al.*, ‘The Role, Challenges, and Support of Pulsenet Laboratories in Detecting Foodborne Disease Outbreaks’, *Public Health Reports*, 2010, 125, 57–62, 59–60.

243 S. L. Inhorn *et al.*, ‘The State Public Health Laboratory System’, *Public Health Reports*, 2010, 125, 4–17.

244 Rosner and Markowitz, *Are We Ready?*, 146.

245 S. J. Schrag *et al.*, ‘SARS Surveillance during Emergency Public Health Response, United States, March–July 2003’, *Emerging Infectious Diseases*, 2004, 10, 185-194, 192.

246 A. S. Khan, ‘Public Health Preparedness And Response In The USA Since 9/11: A National Health Security Imperative’, *The Lancet*, 2011, 378, 953–56, 953-6.

247 Ibid., 955; Rosner and Markowitz, *Are we ready?*, 85.

248 Rosner and Markowitz, *Are we ready?*, 150–54.

249
*Ibid*., 68-9; 74.

250
*Ibid*., 81.

251
*Ibid*., 85.

252 Boxrud *et al.*, ‘The Role, Challenges, and Support of Pulsenet Laboratories in Detecting Foodborne Disease Outbreaks’, 60–1; D. A. Draper, R. E. Hurley, and J. R. Lauer, ‘Public Health Workforce Shortages Imperil Nation's Health’, *Res Brief*, 2008, 4, 1–8.

253 APHL, *Lessons from a Virus. Public Health Laboratories Respond to the H1N1 Pandemic: Association of Public Health Laboratories* (APHL, 2011), 13, 15, 17 and 19.

254
*Ibid*., 25–7, 29.

255
*Ibid*., 71.

256
*Ibid*.

257
*Ibid*., 23, 39, and 55-64.

258
*Ibid*., 40.

259 A. S. Khan, ‘Public Health Preparedness and Response in the USA Since 9/11: A National Health Security Imperative’, 955.

260
*Ibid*.

261 M. L. Wilson, S. Gradus, S. J. Zimmerman, ‘The Role of Local Public Health Laboratories’, 121-22; Inconsistent state reporting and delays also impeded federal COVID-19 responses; *Tracking COVID-19 in the United States. From Information Catastrophe to Empowered Communities* (Vital Strategies—Resolve to Save Lives, 2020).

262 J. C. Ridderhof and B. W. Wilcke Jr, ‘Public Health Laboratory Systems: At the Crossroads’, *Public Health Reports*, 2013, 128, 1–6; C. Loring *et al.*, ‘Using Fee-for-Service Testing To Generate Revenue for the 21st Century Public Health Laboratory’, *Public Health Reports*, 2013, 128, 97–104; M.-B. Malcarney, N. Seiler, and K. Horton, ‘Public Health Laboratories and the Affordable Care Act: What the New Health-Care System Means for Public Health Preparedness’, *Public Health Reports*, 2015, 130, 543–46.

263 D. U. Himmelstein and S. Woolhandler, ‘Public Health’s Falling Share of US Health Spending’, *American Journal of Public Health*, 2016, 106, 56–57.

264 C. L. Richards, M. F. Iademarco, and T. C. Anderson, ‘A New Strategy for Public Health Surveillance at CDC: Improving National Surveillance Activities and Outcomes’, *Public Health Reports*, 2014, 129, 472–76; N. Maddox, ‘100 Years of Influenza: Will Public Health Be Ready for the Next Pandemic’, *APHL Lab-Matters*, Winter (2018).

265 Himmelstein and Woolhandler, ‘Public Health’s Falling Share of US Health Spending’; ‘Time to Welcome the Next Generation of PHL Scientists’, *Lab-Matters* Fall (2018); ‘Invisible No More: Sharing the Value of Health Laboratories’, *APHL Lab-Matters* Summer (2017); L. Alesse, ‘Did Trump Try To Cut the CDC’s Budget As Democrats Claim? Analysis’, *ABC News*, 29 February 2020.

266 ‘Gesetz zur Verhütung und Bekämpfung von Infektionskrankheiten beim Menschen (Infektionsschutzgesetz—IfSG)’, in *Bundesgesetzblatt I*, 2000, 1045; A. Wirtz *et al.*, ‘Rolle der Gesundheitsbehörden der Länder bei der Verhütung und Bekämpfung von Infektionskrankheiten’, *Bundesgesundheitsblatt-Gesundheitsforschung-Gesundheitsschutz*, 2005, 48, 971–78; J. Dreesman and J. Benzler, ‘Surveillance übertragbarer Krankheiten auf der Grundlage des Infektionsschutzgesetzes in Deutschland durch den öffentlichen Gesundheitsdienst’, *Bundesgesundheitsblatt-Gesundheitsforschung-Gesundheitsschutz*, 2005, 48, 979–89; Plümer, ‘Germany’, 35-47.

267 Busse and Blümel, *Germany: Health System Review*, 181–2.

268
*Infektionsepidemiologisches Jahrbuch meldepflichtiger Krankheiten für 2001* (Berlin: Robert Koch Institut, 2002), 11.

269 G. Krause *et al.*, ‘SurvNet Electronic Surveillance System For Infectious Disease Outbreaks, Germany’, *Emerging Infectious Diseases*, 2007, 13, 1548-1555.

270
*Influenza pandemic preparedness plan* (Geneva: WHO, 1999); W.H. Haas, ‘Principles of epidemic emergency preparedness planning: the example of the German Influenza Pandemic Preparedness Plan’, *Bundesgeshundheitsblatt, Gesundheitsforschung, Gesundheitsschutz*, 2005, 48, 1020–1027; M. Dierke. *Deutsches Elektronisches Melde- und Informationssystem für den Infektionsschutz* (Berlin: RKI, 2017).

271 Busse and Blümel, *Germany: Health System Review*, xxvii; Wirtz *et al.* ‘Rolle der Gesundheitsbehörden der Länder bei der Verhütung und Bekämpfung von Infektionskrankheiten’.

272 Plümer, ‘Germany’, 40.

273 A. Graf, ‘Was Tests für alle wirklich bringen würden’, *Zeit Online*, 30 August 2020.

274 L. Wieler, U. Rexroth, and R. Gottschalk, ‘Emerging COVID-19 Success Story: Germany’s Strong Enabling Environment’, *Our World in Data*, published online first: 30 June 2020.

275 C. Smyth, ‘NHS Fails To Cope With Bodies in Flu Pandemic Test’, *The Times*, 27 December 2016.

276
*Global Health Security Index*; *A World At Risk. Annual Report on Global Preparedness for Health Emergencies* (Nuclear Threat Initiative, 2019); *Pandemic Preparedness Financing—Status Update* (Washington DC: World Bank Group, 2019); see also *WHO Joint External Evaluation of IHR core capacities* evaluations on IRIS.

277 Crosby *et al.*, ‘All Bets Are Off For Measuring Pandemic Preparedness’.

278 The genre of the elite disease detective has been popularised by journalists like Laurie Garrett and Maryn McKenna but has also featured in movies like the *Hot Zone*, the CDC has collated fictional, nonfictional, and articles describing its epidemic intelligence service; CDC, *Telling the Story of EIS, CDC, and Epidemiology*, 20 October 2021. https://www.cdc.gov/eis/downloads/eis-telling-the-story.pdf (accessed 26 April 2022); regarding reporting in Britain see, for example, Sarah Bosely, ‘Has Everyone in Kent Gone To an Illegal Rave?’: On the Variant Trail with the COVID Detectives’, *Guardian*, 03 April 2021; Holger Dambeck, ‘Christian Drosten ist Deutschlands gefragtester Corona-Experte’, *Spiegel*, 23 December 2020.

279 WHO, *WHO Hub for Pandemic and Epidemic Intelligence. Better Data. Better Analytics. Better Decisions* (Geneva: WHO, 2021); UKHSA, Press Release: UK Health Security Agency launches with a Relentless Focus on Keeping the Nation Safe, 01 October 2021. https://www.gov.uk/government/news/uk-health-security-agency-launches-with-a-relentless-focus-on-keeping-the-nation-safe, (accessed 26 April 2022); S. G. Stolberg, ‘Biden’s 10-Year Pandemic Preparedness Plan Includes a Full-Time ‘Mission Control’ Office’, *New York Times*, 03 September 2021.

## Notes

Claas Kirchhelle is a Lecturer at University College Dublin, Ireland. Supported by a Wellcome Trust University Award, his research explores the international surveillance, collection and control of microbial pathogens as well as the history of pharmaceutical research and development.

